# Pathogen spillover driven by rapid changes in bat ecology

**DOI:** 10.1038/s41586-022-05506-2

**Published:** 2022-11-16

**Authors:** Peggy Eby, Alison J. Peel, Andrew Hoegh, Wyatt Madden, John R. Giles, Peter J. Hudson, Raina K. Plowright

**Affiliations:** 1 School of Biological, Earth, and Environmental Sciences, University of New South Wales, Sydney, New South Wales Australia; 2 Centre for Planetary Health and Food Security, Griffith University, Nathan, Queensland Australia; 3 Center for Large Landscape Conservation, Bozeman, MT USA; 4 Department of Mathematical Sciences, Montana State University, Bozeman, MT USA; 5 Department of Microbiology and Cell Biology, Montana State University, Bozeman, MT USA; 6 Department of Epidemiology, Johns Hopkins Bloomberg School of Public Health, Baltimore, MD USA; 7 Center for Infectious Disease Dynamics, Pennsylvania State University, State College, PA USA; 8 Department of Public and Ecosystem Health, Cornell University, Ithaca, NY USA; 9 Present Address: Department of Biostatistics and Bioinformatics, Rollins School of Public Health, Emory University, Atlanta, GA USA; 10 Present Address: Institute for Health Metrics and Evaluation, University of Washington, Seattle, WA USA

**Keywords:** Ecological epidemiology, Conservation biology

## Abstract

During recent decades, pathogens that originated in bats have become an increasing public health concern. A major challenge is to identify how those pathogens spill over into human populations to generate a pandemic threat^1^. Many correlational studies associate spillover with changes in land use or other anthropogenic stressors^2,3^, although the mechanisms underlying the observed correlations have not been identified^4^. One limitation is the lack of spatially and temporally explicit data on multiple spillovers, and on the connections among spillovers, reservoir host ecology and behaviour and viral dynamics. We present 25 years of data on land-use change, bat behaviour and spillover of Hendra virus from Pteropodid bats to horses in subtropical Australia. These data show that bats are responding to environmental change by persistently adopting behaviours that were previously transient responses to nutritional stress. Interactions between land-use change and climate now lead to persistent bat residency in agricultural areas, where periodic food shortages drive clusters of spillovers. Pulses of winter flowering of trees in remnant forests appeared to prevent spillover. We developed integrative Bayesian network models based on these phenomena that accurately predicted the presence or absence of clusters of spillovers in each of the 25 years. Our long-term study identifies the mechanistic connections between habitat loss, climate and increased spillover risk. It provides a framework for examining causes of bat virus spillover and for developing ecological countermeasures to prevent pandemics.

## Abstract (web summary)

A study reveals how land-use change and climate interact to drive the spillover of a zoonotic virus, and identifies an ecological mechanism that prevents spillover.

## Main

Zoonotic spillover is the transmission of a pathogen from a non-human vertebrate to a human^1^. Spillovers of viruses from bats have resulted in the emergence and spread of viruses in the human population. For example, SARS-CoV-2, SARS-CoV-1, Nipah and Hendra viruses have caused human mortalities, sometimes after transmission through an intermediate host^5^. Spillover of viruses from wildlife to humans has been correlated with land-use change through studies that associate land use, occurrence of spillover and presence of reservoir hosts, but without data that reveal the mechanisms^1^. In this long-term study, we observed rapid changes in bat behaviour that coincided with the emergence of Hendra virus. We found that bats were responding to environmental change by persistently behaving in ways that were previously observed as temporary responses to climate-driven food shortages. We propose that these behavioural shifts increased spillover risk by increasing contact of bats with domestic horses, the intermediate hosts from which Hendra virus spills over into humans, and by increasing viral shedding from bat populations that have established outside their normal winter range^6^. We developed and applied a Bayesian hierarchical network model to our 25 years of data on reservoir host ecology, behaviour and spillover events (Data Index in ref. ^7^). We identified distal and proximal drivers of links between habitat loss, climate and spillover, and predicted the risk of Hendra virus spillover in space and time. The Hendra virus system illustrates a suite of ecological connections that contribute to land-use-induced spillover of this pathogen: interactions between land-use change and climate altered the behaviour of wildlife reservoir hosts, increasing their proximity to domestic or human recipient hosts. We propose that this phenomenon, coupled with stressors that drive increased pathogen excretion^6^, leads to pathogen spillover.

**Fig. 1:**
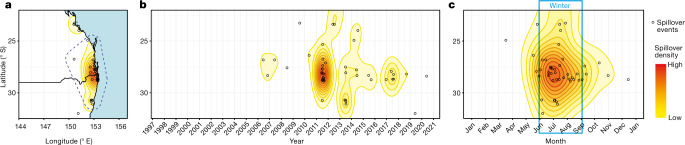
Temporal and spatial distribution of documented Hendra virus spillovers to horses in the Australian subtropics from 1996 to 2020. **a**, Distribution of spillovers across subtropical eastern Australia from 1996 to present. The dotted blue line denotes our study area. **b**, Hendra virus spillover by date of horse fatality from 1996 to present. Spillover events cluster in space in some years. **c**, Hendra virus spillover by month. In the subtropics, spillovers occur primarily during the Austral winter. The base-map was obtained from the Australian Bureau of Statistics digital boundary files (www.abs.gov.au).

Australian flying foxes (*Pteropus* spp., fruit bats) are reservoir hosts of Hendra virus, a henipavirus in the family Paramyxoviridae that does not cause discernible disease in bats, but has a case fatality rate of 75% in horses (84 fatalities documented) and 57% in humans (four fatalities documented)^8^. Among subtropical Pteropodids, *Pteropus alecto* (black flying fox) is the species most likely to excrete Hendra virus^9^. Infected bats feeding in horse paddocks shed the virus in excreta or spats that horses contact when grazing, leading to infection^9,10^. Humans are exposed through infected horses^8^. Hendra virus has probably circulated in bats far longer than Europeans have occupied Australia^11^, yet Hendra spillover was not identified until 1994. After approximately 2006, the frequency of Hendra virus spillovers increased^10^. Forty one of the 63 spillovers documented to January 2021 occurred in subtropical eastern Australia^12^. The majority of spillovers occurred during the Austral winter and were clustered in space and time (Fig. 1 and Supplementary Information Section 1).

Our research focused on spillover to horses in the Australian subtropics, where *Pteropus* spp. primarily feed on nectar. Historically, the bats were nomadic, moving among roost sites to track ephemeral pulses of flowering by *Eucalyptus* spp. over hundreds of kilometres^13–15^; continuous occupancy of roosts was uncommon^13^. During summer, many tree species that are abundant and widely distributed provide food for bats^16^. During winter, few tree species provide food for bats, and the naturally limited distribution and abundance of these trees^16^ has been further restricted by clearing for urban development and agriculture^17,18^. Pulses of mass flowering in remnant forests leads to a high proportion of the bat population becoming concentrated in small areas, typically in coastal lowlands^13^. Loss of these winter food sources can have severe impacts on the bat population^16,17^. When trees that provide food in winter or spring do not flower, as occurs every 1 to 4 years owing to variation in temperature and rainfall^19,20^, bats experience brief (typically 3 to 12 week) food shortages. They respond to the shortages by roosting in small groups (population fission) close to reliable but often suboptimal food in urban gardens and agricultural areas (for example, fruit from ornamental, commercial or weed species)^13,17,21–23^. In the past, these behavioural responses persisted only for the duration of acute food stress, and bats returned to nomadism and nectivory when the food shortage abated^22,24,25^.

**Extended Data Fig. 1:**
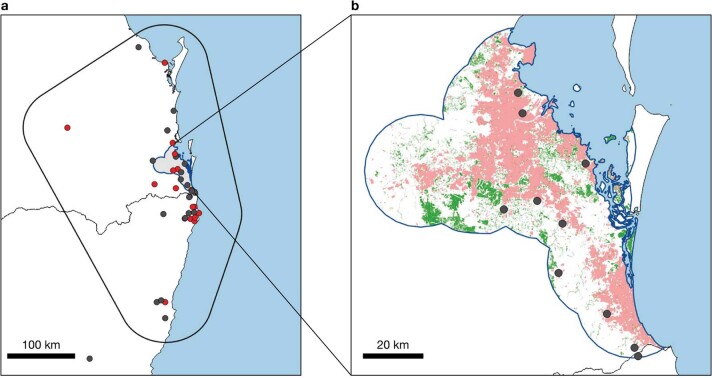
Boundaries of the Hendra virus study area and Southeast Queensland study area with locations of Hendra virus spillovers to horses indicated. **(a)** The full study area with locations of subtropical Hendra virus spillovers (circles, Database A)^12^; **(b)** The far Southeast Queensland (SEQ) study area showing the Hendra virus spillovers documented within the boundary (circles, Database A)^12^; and the 2018 distributions of built land use (light red, Supplementary Information 9) and extant winter foraging habitat for flying foxes (green, Supplementary Information 12). Base-maps were obtained from the Australian Bureau of Statistics Digital boundary files (www.abs.gov.au).

To investigate the relationships between land-use change, changes in bat behaviour and spillover, we collected empirical data from 1996 to 2020 within an area bounded by the locations of spillovers in the subtropics (Fig. 1a, Extended Data Fig. 1a and Supplementary Information Section 1). These data include the location and timing of Hendra virus spillovers (Dataset A in ref. ^12^), locations and occupancy of the roosts (Datasets B,C)^26,27^, characteristics of the roosts and foraging areas around the roosts (Datasets B,C,I)^26–28^, climate (Dataset D in the Data Index)^7^, nectar shortages (Dataset E)^29^, measures of bat fitness (Dataset F and G)^30,31^, flowering pulses during winter (Dataset J)^32^ and loss of winter foraging habitat (Datasets K,L and M in the Data Index^7^ and Supplementary Sections  1–13).

**Fig. 2:**
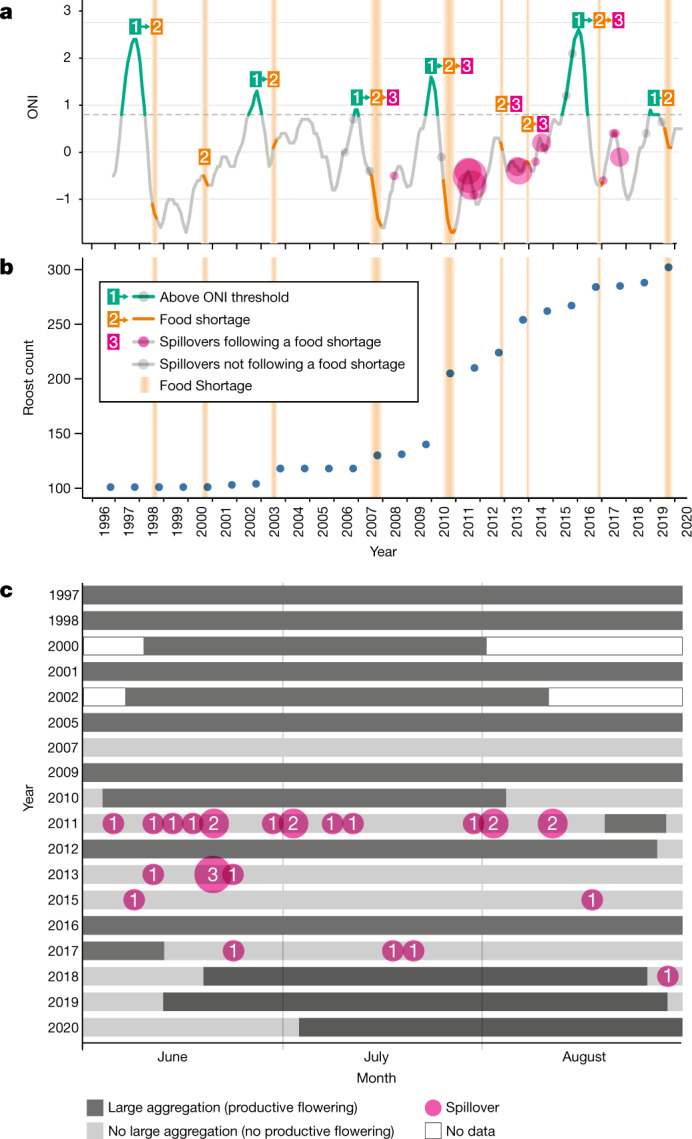
Relationships between climate, periods of nutritional and energetic stress for bats and Hendra virus spillovers. **a**, Temporal associations among the ONI, acute food shortages (identified by apiarists) and winter spillovers. A strong El Niño event (ONI > 0.8) consistently led to food shortages the following winter or spring, although food shortages can occur independently of ONI. During the early study period (1996 to 2002), food shortages did not lead to Hendra virus spillovers; during the period of rapid change, when bat populations were fissioning into urban and agricultural areas, food shortages led to Hendra virus spillovers during the following winter. **b**, Number of roosts in the study area. **c**, Timing of winter spillover events in relation to the presence of large aggregations (at least 100,000 bats) and productive flowering of diet species in southeast Australia during winter (June to August). Each row indicates a winter for which data were available. Data are missing for winter aggregations for 1999, 2003, 2004, 2006, 2008 and 2014 (Supplementary Table 5a).

From 1996 until approximately 2002, roosting and foraging behaviours were stable, and no Hendra virus spillovers were detected (Figs. 1 and 2a,b). From approximately 2003 until 2020, bat behaviour and the incidence of spillovers changed rapidly: the number of roosts tripled, and 40 spillovers were detected (Supplementary Information Sections 1 and 3 and Figs. 1 and 2). From 2006 to 2020, spillovers were detected in 80% of years, and 75% of spillovers occurred in annual clusters of three or more (Fig. 1). Neither a change in the definition of a Hendra virus case in horses in 2008, nor the availability of a horse vaccine in late 2012, explain the observed pattern of spillover events (Supplementary Information Section 1).

**Extended Data Fig. 2:**
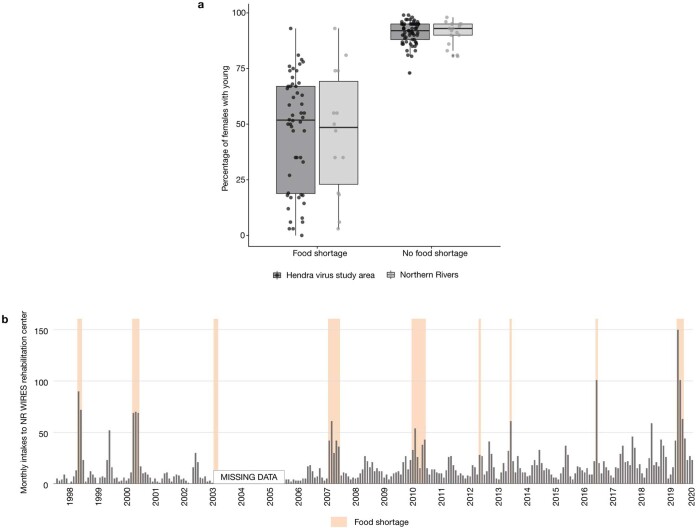
Measures of flying fox fitness identified as important proxies of food shortage in regression tree model. **(a)** index of reproductive output: annual estimates of the percentage of adult females occupying roosts in the Northern Rivers (NR) region (light grey) and throughout the Hendra virus study area (black) carrying live young pre-weaning; data are grouped according to whether a period of nectar shortage was recorded by apiarists during the winter or spring associated with that birth cohort (n = 9 years) versus cohorts when a nectar shortage was not recorded (n = 14 years). Data acquired in years of food shortage were widely dispersed relative to highly clustered data acquired in years without food shortage. Patterns of data distribution were consistent between the NR region and the full study area. Box plots show median values, upper and lower quartiles and all data points. **(b)** monthly counts of admissions to Northern Rivers WIRES wildlife rehabilitation center with months classified as nectar shortage by apiary data (indicated by orange bars). No intake records were available from June 2003 through December 2005. The bounds of the box correspond to the 25^th^ and 75^th^ percentiles, the middle dash is the median, and the whiskers extend to the largest value no further than 1.5 times the interquartile range from the 25^th^ or 75^th^ percentile.

**Extended Data Fig. 3:**
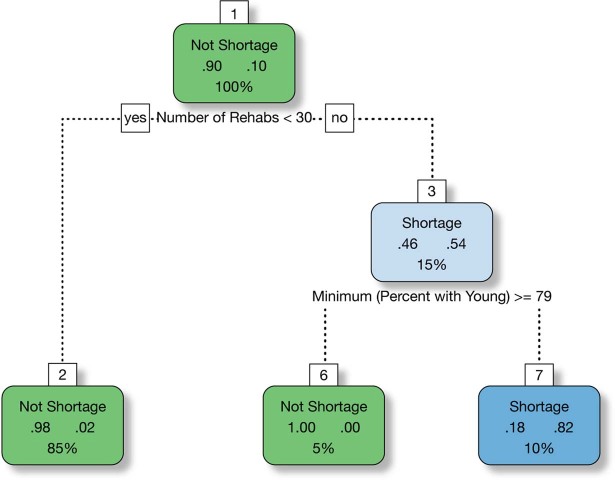
Regression tree fit for identifying flying fox food shortages (as assessed by absence or very low nectar production). Food shortages were predicted when monthly flying fox intakes into wildlife rehabilitation centers were ≥30 individuals and minimum proportion of female flying foxes with young pre-weaning was <79%.

We used nectar productivity data from commercial apiarists and measures of flying fox fitness to identify nine winter and spring food shortages during the study period (Supplementary Information Section 7 and Fig. 2a,b). During food shortages, the absence of nectar was associated with a sharp increase in the number of bats admitted into wildlife rehabilitation centres (at least 30 animals in a month) and a low percentage of lactating females with pre-weaning young (<79%; Supplementary Information Section 7 (ref. ^33^) and Extended Data Figs. 2 and 3). Food shortages followed all strong El Niño events (Oceanic Niño Index ≥ 0.8), and also occurred independently of El Niño events (Fig. 2a).

**Fig. 3:**
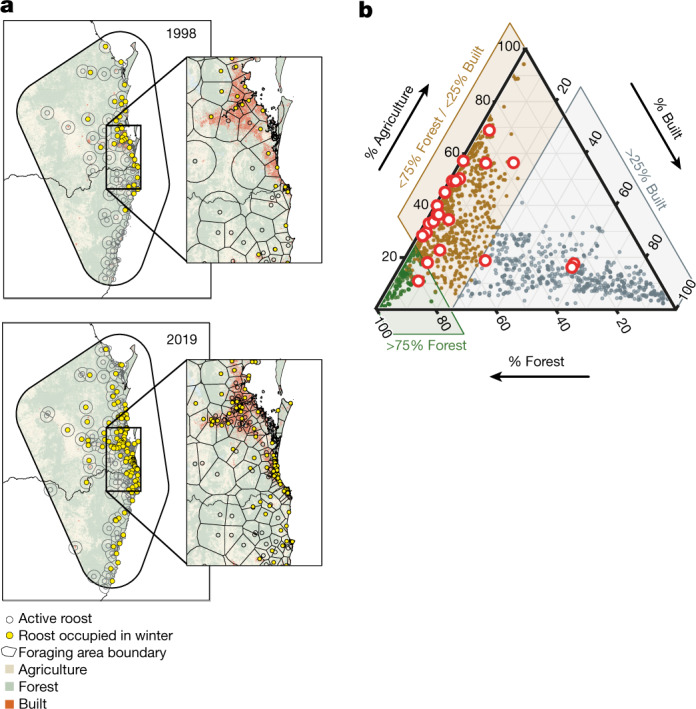
The distribution of *Pteropus alecto* roosts during winter. **a**, Expansion of the number and distribution of roosts occupied by *Pteropus alecto* during winter from 1998 to 2019. The base-map was obtained from the Australian Bureau of Statistics digital boundary files (www.abs.gov.au). **b**, Proportion of the foraging area surrounding roosts that was classified as built, forested or agricultural (Supplementary Information 13). The red circles indicate roosts that were the sources of winter spillovers.

**Extended Data Fig. 4:**
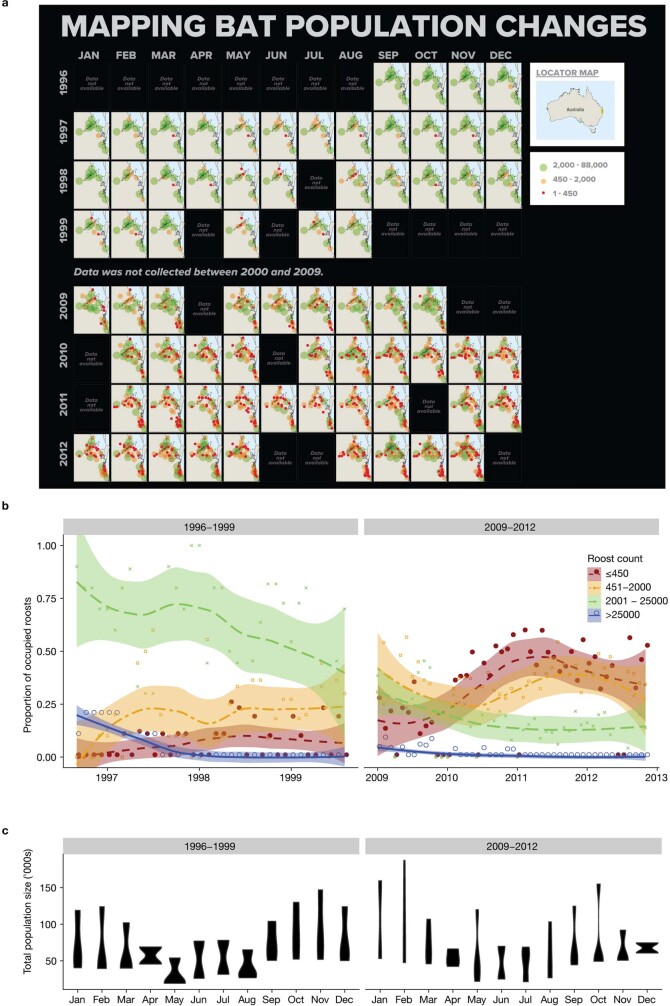
Changes through time in the number and estimated population size of roosts occupied by black flying foxes *Pteropus alecto* in the SEQ study area. Censuses of roosts were conducted monthly in two time periods: T1 = 1996–1999 and T2 = 2009–2012. **(a)** Monthly maps of roost locations and population size grouped in three quantiles. The base-map was obtained from the Australian Bureau of Statistics Digital boundary files (www.abs.gov.au). **(b)** An additional population size category (>25,000) was added to illustrate change in large roosts. The line is a LOESS best fit and gray shading indicates 95% confidence intervals. **(c)**. Total population estimates of black flying foxes (all roosts combined) grouped by month and year and summarized in violin plots. The number and locations of roosts was stable in T1 (n = 12) and increased from 34 to 80 in T2, although total estimates of black flying foxes in the study area remained stable.

**Extended Data Fig. 5:**
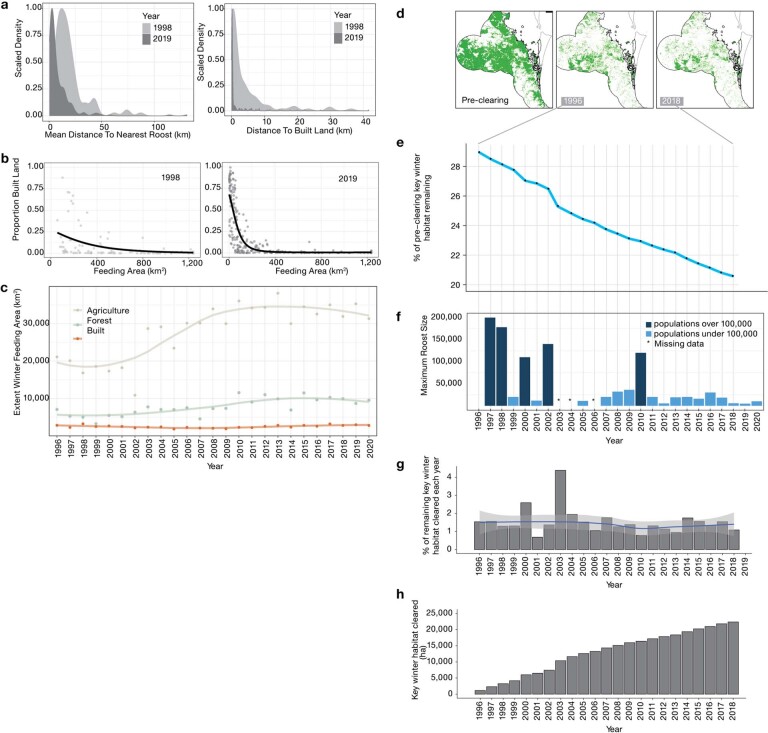
Spatial and temporal changes in the roosting and foraging behaviors of *Pteropus* bats during the study period (1996–2019 comparisons); and coincident decline in the extent of winter habitat in the SEQ study area. **(a)** Mean distance between nearest neighboring roosts and the distance between roosts and developed areas decreased. **(b)** Roost occupancy in built land in metropolitan centers increased; smaller foraging areas contained a higher proportion of urban land; **(c)** Annual change in the area of different land-cover types within the total winter foraging range of *P. alecto*. Data are the summed total of the area assigned to each land use type in the foraging areas of roosts occupied in winter. The total area of land in the winter foraging range increased over time, primarily as a result of expansion into agricultural areas. Although the number of winter roosts in urban areas increased substantially, the total area of built land in combined foraging areas did not. **(d)** Distribution of key winter habitat in the southeast Queensland study area (SEQ): pre-clearing, at the start of the study in 1996 and at the end of the study in 2018. **(e)** Percentage of pre-clearing winter habitat remaining (note that the y-axis is compressed). **(f)** Abundance of bats in the largest winter roosts within SEQ. Aggregations ≥100,000 were common pre-2005, rare after 2005, and none occurred from 2011 to 2020. **(g)** Annual rate of clearing of key winter habitat in the SEQ study area 1996–2018, the line is a LOESS best fit. Gray shading indicates 95% confidence intervals and **(h)** Cumulative area cleared (ha).

Early in the study period, the number of roosts was stable. Although food shortages occurred, the bat population fission events were brief (weeks), bats quickly returned to nomadism when the shortage abated and the fission events were not captured in the roost counts. Following this, the number of roosts increased in steps that coincided with food shortages (2003, 2007 and 2010) and then continued to increase steadily (Fig. 2b). As the number of roosts increased, bats roosted in smaller groups that were closer together and fed within smaller areas, and new roosts formed in locations that increased access to anthropogenic foods in agricultural and urban areas (Fig. 3 and Extended Data Figs. 4 and 5a–c). We infer that fissioning of bat populations into smaller groups near anthropogenic food reduces the energetic costs of foraging and allows the bats to mitigate effects of nutritional and energetic stress^25^. Whereas in the past, the behavioural response of bats to food shortages (population fission) persisted only for the duration of acute food stress^22,24,25^, we observed the behavioural response becoming persistent (Extended Data Fig. 4). To assess the mechanisms associated with the rapid change in bat behaviour, we examined loss of winter foraging habitat across far Southeast Queensland (Supplementary Information 12 and Extended Data Fig. 1b). Before European settlement, winter habitat was extensive in this region (Extended Data Fig. 5d). More than 70% of the forest that provided winter habitat was cleared before 1996, and clearing continued at a constant rate (Extended Data Fig. 5e,g,h). In four of the six years from 1996 to approximately 2002, during which bat behaviour was consistent and no spillover occurred, we recorded aggregations of at least 100,000 nomadic bats associated with mass pulses of flowering in winter habitat in this region (Extended Data Fig. 5f). By 2018, nearly a third of the habitat that was present in 1996 had been cleared (Extended Data Fig. 5e); the number of roost sites had increased fivefold (Extended Data Fig. 4), with 87% of new roosts forming in urban areas. By 2020, winter aggregations of at least 100,000 in far Southeast Queensland had been recorded once in 14 years (Extended Data Fig. 5f). Although bats relocated to both urban and agricultural areas, most spillovers (86%) occurred in agricultural areas (Fig. 3b), presumably because horses were present at higher densities in agricultural areas than in urban areas.

**Fig. 4:**
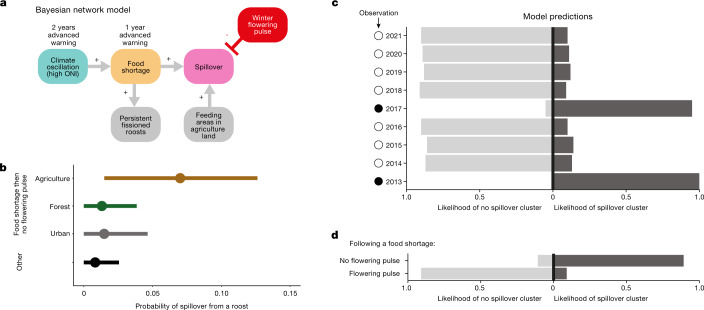
Prediction of spillover on the basis of bat ecology and ecological conditions. **a**, Structure of the Bayesian network model: strong El Niño events preceded food shortages that were associated with population fissioning and formation of persistent roosts in agricultural and urban areas. Spillover risk was greatest when *Pteropus alecto* fed in agricultural areas during a winter that followed an acute food shortage. The presence of a pulse of winter flowering that attracted large aggregations of bats mitigated spillover risk. Time delays between high ONI, food shortages and spillover allow advanced prediction, although winter-flowering pulses that mitigate spillover could not be predicted in advance. **b**, Probability of a spillover at the roost level given scenarios of land use under the condition of a food shortage followed by no winter-flowering pulse. Other, collates all other scenarios of the presence and absence of food shortages and flowering pulses. The circles are maximum a posteriori point estimates, and the bars are 95% highest posterior density credible intervals. **c**, Predicted probability of a cluster of spillovers in each year from 2013 to 2021. Predictions for a given year were made with all observations to that date; information from future years was not incorporated into predictions. In 2017 and 2013, food shortages in the year before and no winter-flowering pulse led to elevated risk of spillover, whereas in the other years, no clusters of spillovers were predicted. All predictions were consistent with the realized data on the absence (open circles) or presence (closed circles) of clusters of spillover. **d**, Predictive probability of a cluster of spillovers in winter 2020, following a food shortage in 2019. A winter-flowering pulse occurred in early July 2020 and no winter spillovers were observed. We predicted that a cluster of spillover events would have occurred if there had not been a flowering pulse.

**Extended Data Fig. 6:**
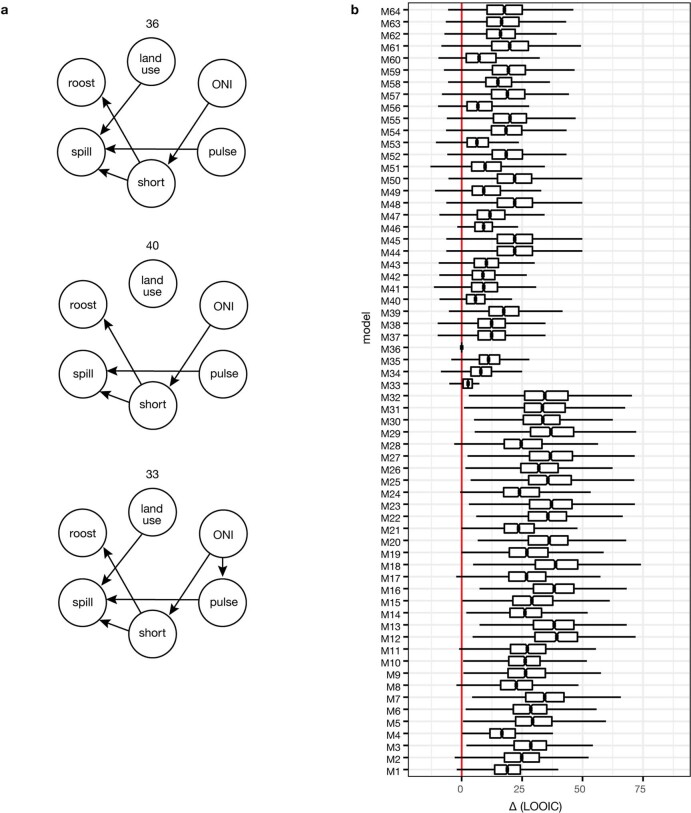
A total of 64 different plausible network models were assessed using expected log pointwise predictive density (ELPD) and leave-one-out (LOO) cross-validation for predictive validation. **(a)** The figure contains the best network, Model 36 (top), plus all of the other networks within 10 leave-one-out information criteria (LOOIC) units of Model 36. The node labels are abbreviated; spill is spillover, roost is roost fissioning, land use is the land use type at the roost level, short is a food shortage in the previous year, pulse is a winter flowering pulse, and ONI is elevated ONI over 0.8 two years prior. **(b)** Distribution of LOOIC values relative to Model 36. For each model, the boxplots (n = 1000 simulations) show the distribution of the difference in LOOIC. M36 is the favored model as the median, and at least the first quartile, are positive for all other models. The bounds of the box correspond to the 25^th^ and 75^th^ percentiles, the middle dash is the median, and the whiskers extend to the largest value no further than 1.5 times the interquartile range from the 25^th^ or 75^th^ percentile.

**Extended Data Fig. 7:**
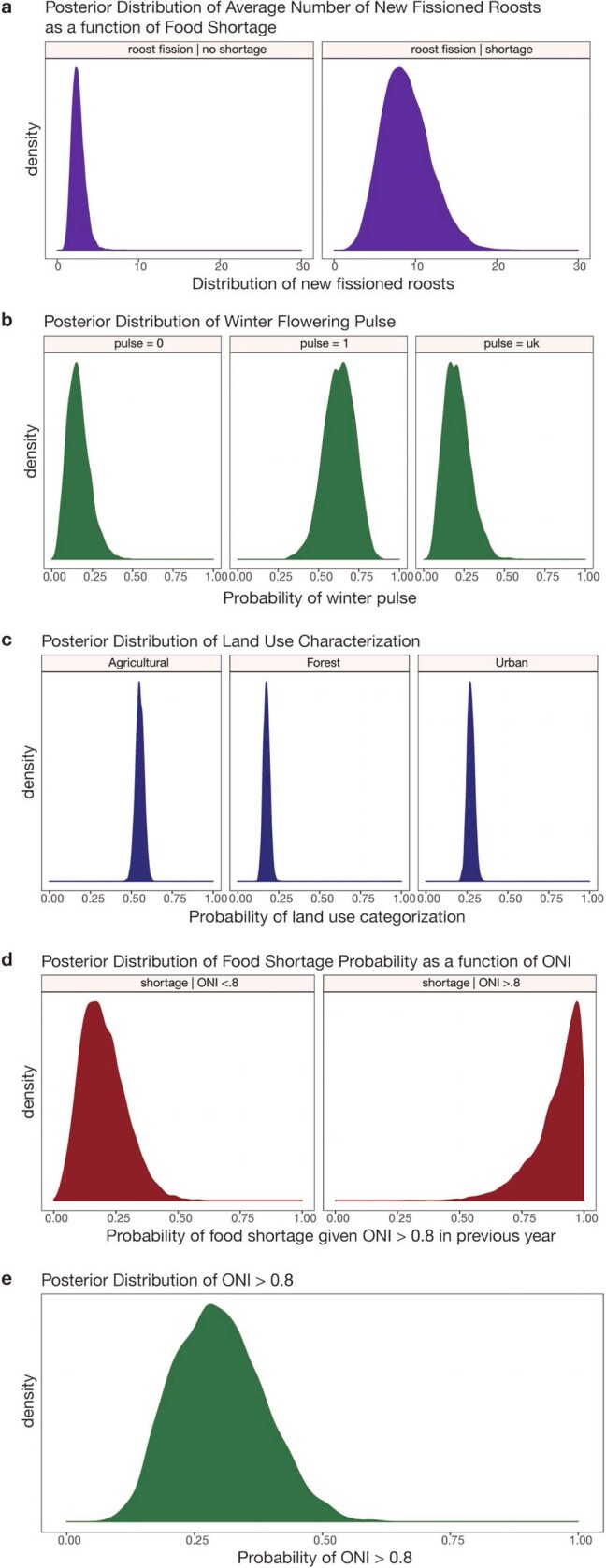
Posterior distributions for model parameters in Bayesian Network Model. **(A)** posterior distribution of new fissioned roosts, conditional on food shortage events. **(B)** posterior distribution for the probability of a winter flower pulse. **(C)** posterior distribution for the probability of a roost having the three different land use characteristics. **(D)** posterior distribution for the probability of a food shortage given elevated ONI values. **(E)** the posterior distribution for the probability of elevated ONI values.

**Extended Data Fig. 8:**
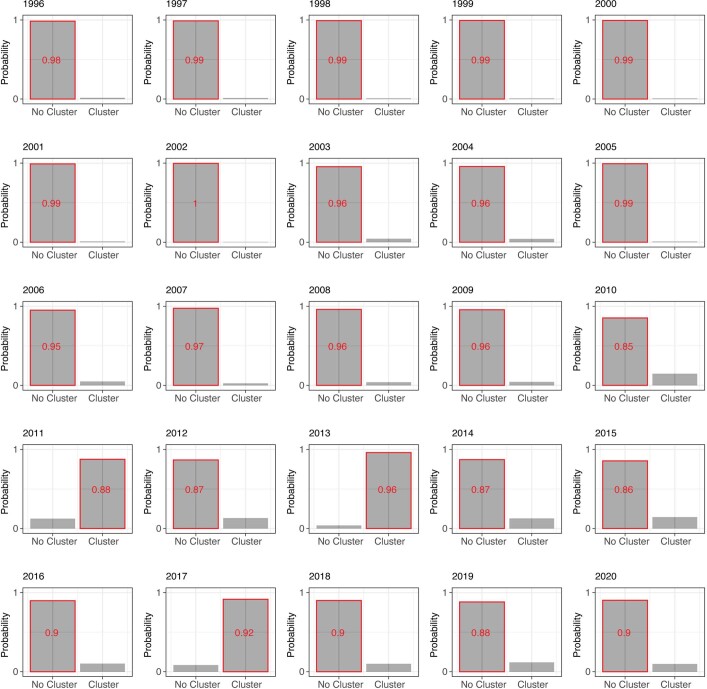
Output from 5-fold cross validation study to assess the ability of our model to predict spillover clusters. The red bars correspond to whether a cluster of spillovers was observed in that year and the grey bars and number correspond to predicted probabilities. For a given year, roost-level predictions are made using models fit on roosts-year combinations from other folds. This approach provides strong evidence of the predictive ability of the model framework as it predicts, with high certainty, the presence or absence of clusters of spillovers in all 25 years.

**Extended Data Fig. 9:**
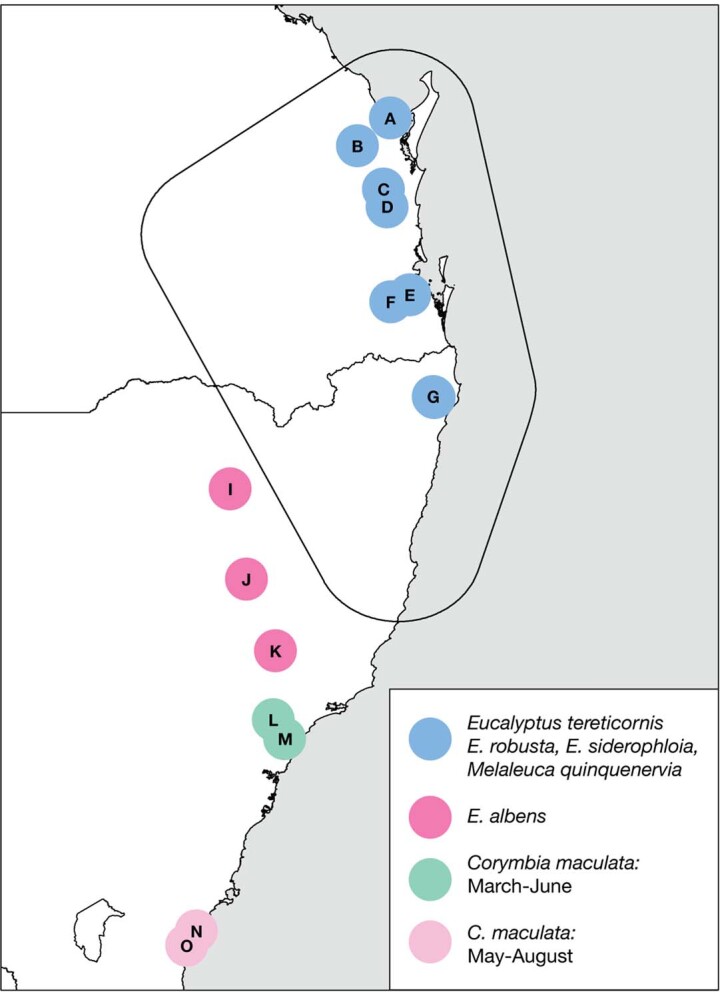
The locations of pulses of winter flowering in southeast Australia 1997–2020 as identified by aggregations ≥100,000 of nomadic grey-headed flying foxes and black flying foxes; and the winter flowering diet plants that occur in local vegetation communities. Winter habitat in the Hendra virus study area primarily comprises a mix of four species, two single species occur in other regions. Latitudinal variations in the flowering phenology of *Corymbia maculata* are indicated. Dates are provided in Dataset J^32^, a key to roost names is provided in Supplementary Table 5B. The base-map was obtained from the Australian Bureau of Statistics Digital boundary files (www.abs.gov.au).

We developed the parsimonious hypothesis that the combination of acute winter/spring food shortages followed by a lack of winter flowering in the following year yields the highest risk of spillover, especially from roosts in agricultural areas. We tested this mechanistic hypothesis with a multiscale Bayesian network model^34^. We included six variables: strong El Niño events (Oceanic Niño Index ≥ 0.8), food shortages, winter-flowering pulses, land cover within the foraging areas of winter colonies of *P. alecto*, population fissioning (establishment of new roosts) and number of Hendra virus spillovers (Supplementary Information Section 13 and Supplementary Fig. 1). We validated the predictive ability of the network model with leave-one-out cross-validation and expected log pointwise predictive density. We verified this by using a leave-one-out cross-validation for model selection with more than 1,000 simulated datasets (Supplementary Information Section 13). The best model supported that spillover clusters occur following a sequence of events over three successive years. First, it indicated that strong El Niño events preceded food shortages, and food shortages coincided with population fissioning (Fig. 4a, Extended Data Figs. 6–8 and Supplementary Tables 7–9). Second, the greatest likelihood of spillover from a given roost was associated with *P. alecto* feeding in agricultural areas during a winter that followed an acute food shortage, but in which there was no pulse of flowering (0.014, 0.126 highest posterior density probability interval; Fig. 4b). By contrast, the presence of a pulse of winter flowering that attracted at least 100,000 bats mitigated spillover risk (0.000, 0.026 probability interval; Fig. 2c and Fig. 4b, Extended Data Fig. 9 and Supplementary Table 5).

**Extended Data Fig. 10:**
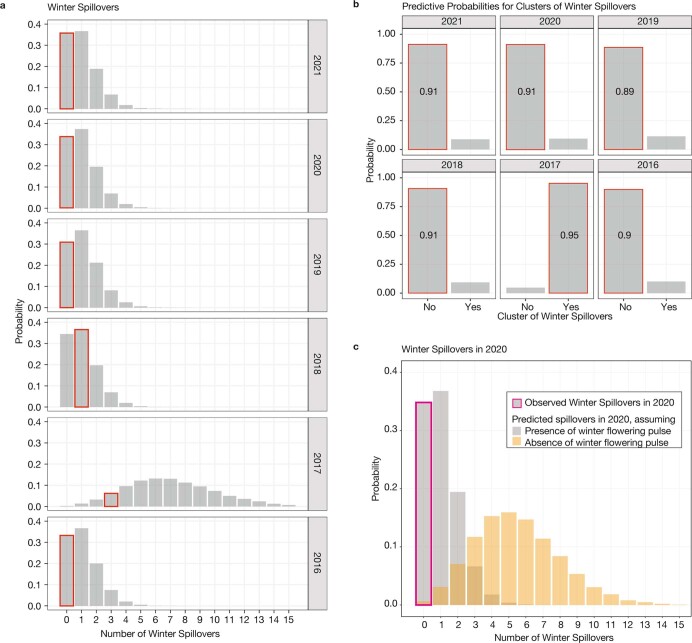
Predicted probabilities for total number and clusters of winter spillovers. **(a)** Predicted probability for total number of winter spillovers (grey bars) and observed number of winter spillovers (red outlines). **(b)** Predicted probability for a cluster of spillovers (grey bars, defined as three or more spillovers) and the observed presence or absence of a cluster of winter spillovers (red outlines). In both figures, predictions are made using all observed data up to that time, but information from future years is not incorporated into predictions. The period of 2016 through 2021 was chosen for probabilistic predictions because enough preceding data allowed predictions. Food shortages were seen preceding years 2017 and 2020, but only the year 2017 included both a food shortage and a lack of a winter flowering pulse. All the predictions are supported by the realized data, which fall in higher mass parts of the distributions in **(a)** and align with predictions with high predicted probabilities in **(b)**. **(c)** Predictive distribution of the total number of spillovers in winter 2020, following a food shortage in 2019. The grey bars indicate the spectrum of probabilistic predictions for spillover, given a winter flowering pulse. A winter flowering pulse did occur in early July 2020 and no winter spillovers were observed (pink outlined bar). Had no flowering pulse occurred, we predicted many more spillover events (yellow bars).

We used the model to make probabilistic predictions of whether a cluster of spillovers (defined as three or more spillovers) would occur in the study area from 1996 to 2021. We used all available information up to a given time point, starting with 2013 (Fig. 4c and Extended Data Fig. 10a,b). For example, to predict a cluster of spillovers in 2020 (Fig. 4d and Extended Data Fig. 10c), we used information collected to the end of 2019. In all cases, model predictions were consistent with observations of the presence or absence of clusters of spillovers in the context of winter flowering. We also verified the predictive ability of our model with fivefold cross-validation; our model accurately predicted the presence or absence of a cluster of spillovers in each of the 25 years in the data (Extended Data Fig. 8).

These data document increasing rates of transmission of a fatal zoonotic virus in a rapidly changing anthropogenic system. Our work suggests that previously transient fissioning of bat populations in response to periods of food shortage has become persistent. This behavioural response leads to the increased use of agricultural and urban areas by bats in winter (Fig. 3). Food shortages occurred throughout the 25-year study period (Fig. 2a,b), probably driven by the lagged effects of climate on *Eucalyptus* flowering^20^. However, spillovers were not associated with food shortages until bats persistently overwintered in agricultural areas in proximity to horses, and winter-flowering pulses became less frequent (Figs. 2c,3 and 4a). We propose that the loss of winter-flowering habitat and consequent decline in the abundance of winter nectar contributes to the persistence of bats in agricultural and urban areas (Supplementary Information Section 12, Fig. 2c and Extended Data Fig. 5d–h). The consequences of more bats in areas with human settlements include not only increased risk of viral spillover from bats to horses to humans, but also increased conflict with humans^35^.

The contemporary association between food shortages, population fission and spillover may be explained by both increased proximity of bats and horses, and physiological stress in novel winter habitats. Bats in roosts that recently formed outside of the historic winter range of the species excreted more Hendra virus in winter, especially after food shortages, than bats in roosts within the historic winter range of the species^6^. Bats in urban and agricultural areas rely on suboptimal food, which may lead to nutritional stress that facilitates viral shedding^36–39^. Nutritional stress has been linked to infection and shedding of Hendra virus^36,39^, coronaviruses^40^ and other viruses in reservoir host systems^41^; moreover, Hendra virus spillover events in the subtropics have been associated with small roosts with limited access to native food^19^. The timing of Hendra virus spillover clusters in winter, months after the food shortages in the previous year, may be owing to the cumulative effects of nutritional stress overlaying high energy requirements in winter (thermoregulation and pregnancy) and scarce resources within suboptimal habitats. Although the time-lag between food shortages and spillover requires further investigation, we suggest that the processes driving spillover of Hendra virus are general phenomena that link land-use change and pathogen emergence: behavioural responses to loss of habitat increase contact with recipient hosts, and physiological and immunological responses to food limitation increase pathogen excretion^4^.

Our data suggest that increasingly rare winter-flowering pulses reduce the risk of spillover. Bats reverted to nomadism and left agricultural and urban areas during pulses of winter flowering in remnant native forest, and spillovers did not occur during these flowering pulses (Fig. 2c). We propose that these pulses of flowering may mitigate zoonotic risk by drawing large numbers of bats (Supplementary Information Section 11 (ref. ^32^)) away from feeding in agricultural areas and, therefore, decreasing contact between bats and horses. This nomadism may also reduce competition for food among the bats that remain in agricultural areas. Understanding these mechanisms requires further work. Nevertheless, the loss of native forest that supports large aggregations of nomadic bats appears to be fundamental to the cascade of events that lead to spillover. An extensive programme of ecological protection and restoration of winter-flowering forests (ecological countermeasures) could be a sustainable, long-term strategy to reduce spillover and protect the health of livestock and humans^42^.

The consistent temporal association of Oceanic Niño Index (ONI) thresholds with food shortages and spillover events allows for the prediction of spillover clusters up to two years in advance through surveillance of climate, or alternatively, one year in advance through surveillance of bat reproduction and bat admissions to wildlife rehabilitation centres (Fig. 4c). However, we cannot as yet predict winter-flowering pulses, and these must be monitored in real time. For example, the conditions leading into winter 2020 suggested that multiple spillover events would occur (Fig. 4d). However, we were unable to predict the pulse of winter flowering that attracted more than 200,000 bats that appears to have prevented winter spillovers. With retrospective information on flowering, our Bayesian network model correctly predicted the absence of a cluster of spillover events in 2020.

Prevention of spillovers requires characterization of the interactions of reservoir and recipient species with their environment in the context of rapid land-use change and climate variability. Monitoring of ecological processes that occur at different temporal extents, including those longer than natural climatic cycles such as El Niño, is essential to understanding the drivers of spillover. Yet long-term data on reservoir hosts, especially bats, are sparse^43^. Many bat species that are the reservoir hosts of zoonotic pathogens depend on ephemeral resources, and occupy ecosystems in which loss of native vegetation, high livestock density and human populations coincide^44–46^. Moreover, some bat species can adapt to human-modified landscapes to mitigate the effects of loss of their native habitat. These species may present greater risks of spillover, as has been observed in other wildlife taxa^47^. We identified key processes connecting land-use change to spillover through behavioural responses of bats to altered food availability. We suggest that behavioural and physiological responses to rapid, human-induced environmental change increased contact between reservoir hosts and recipient hosts, and increased shedding of pathogen^6^ within proximity of recipient hosts. This study, therefore, suggests a general framework for examining causes and potential ways to mitigate bat virus spillover in regions without long-term data on reservoir hosts.

### Reporting summary

Further information on research design is available in the Nature Portfolio Reporting Summary linked to this article.

## Online content

Any methods, additional references, Nature Portfolio reporting summaries, source data, extended data, supplementary information, acknowledgements, peer review information; details of author contributions and competing interests; and statements of data and code availability are available at 10.1038/s41586-022-05506-2.

## Supplementary information

Supplementary Information This file contains details of the methods used to generate and analyse data for this study and extended supporting material that assists with the interpretation of long-term datasets. The file includes links to a Data Index that provides an overview of the datasets used in the study, descriptions of data fields, data sources, availability of data, the analyses the datasets contributed to, and the Supplementary Information sections where those analyses are described. Links are also provided to access the compiled input data for models and their output. The file includes methods for the multiscale Bayesian network model, methods used to generate data for the model and context for interpreting the results.

Reporting Summary

## Data Availability

The datasets generated and analysed during the current study are available in the Cornell University eCommons Digital Repository or they are available as open access files. The URLs are provided in the Data Index 10.7298/pjjb-3360, with the exception of records from commercial apiarists (Supplementary Information Section 7) that are constrained by commercial in-confidence considerations. Dataset URLs: Dataset A, 10.7298/3dbp-t721; Dataset B, 10.7298/kdht-sp38; Dataset C, 10.7298/ajmw-mp18; Dataset E, 10.7298/tb5p-dr98; Dataset F, 10.7298/j3q2-gw32; Dataset G, 10.7298/3vha-5m37; Dataset I, 10.7298/x71e-c660; Dataset J, 10.7298/rmhz-dc23.

## Code availability

Code generated during the current study, and the input data for the models, are available in the Supplementary Information and Cornell University eCommons Digital Repository: 10.7298/rbde-cy49; 10.7298/y0nr-e545.
