# Supplementary material for: Pathogen spillover driven by rapid changes in bat ecology
Source: Nature. 2022 Nov 16;613(7943):340–4. doi: 10.1038/s41586-022-05506-2 (PMC9768785; doi:10.1038/s41586-022-05506-2)
Supplement: Supplementary file 1 — This file contains details of the methods used to generate and analyse data for this study and extended supporting material that assists with the interpretation of long-term datasets. The file includes links to a Data Index that provides an overview of the datasets used in the study, descriptions of data fields, data sources, availability of data, the analyses the datasets contributed to, and the Supplementary Information sections where those analyses are described. Links are also provided to access the compiled input data for models and their output. The file includes methods for the multiscale Bayesian network model, methods used to generate data for the model and context for interpreting the results. [file 41586_2022_5506_MOESM1_ESM.pdf]

nature portfolio

https://doi.org/10.1038/s41586-022-05506-2

Supplementary information

Pathogen spillover driven by rapid changes in bat ecology

In the format provided by the authors and unedited

Supplementary Information

Pathogen spillover driven by rapid changes in bat ecology

Peggy Eby[1,2,3], Alison J. Peel[2], Andrew Hoegh[4], Wyatt Madden[5,†], John R. Giles[6,††], Peter J. Hudson[7], Raina K. Plowright[5,8]*

[1] School of Biological, Earth, and Environmental Sciences, University of New South Wales, Sydney, New South Wales, Australia

[2] Centre for Planetary Health and Food Security, Griffith University, Nathan, Queensland, Australia

[3] Center for Large Landscape Conservation Bozeman, Montana, USA

[4] Department of Mathematical Sciences, Montana State University, Bozeman, Montana, USA

[5] Department of Microbiology and Immunology, Montana State University

Bozeman, Montana, USA

[6] Johns Hopkins University Bloomberg School of Public Health, Department of Epidemiology Baltimore, Maryland, USA

[7] Center for Infectious Disease Dynamics, Pennsylvania State University State College, Pennsylvania, USA

[8]Department of Public and Ecosystem Health, Cornell University, New York, USA.

[†]Current address: Department of Biostatistics and Bioinformatics, Rollins School of Public Health, Emory University, Atlanta, GA, USA

[††]Current address Institute for Health Metrics and Evaluation, University of Washington, Seattle, WA, USA

∗To whom correspondence should be addressed;

E-mail: raina.plowright@cornell.edu

Supplementary Information

This PDF File contains supplemental methods, results, tables and figures under the following section headings

1. Register of Hendra virus spillover events in Australia 4
2. Study areas 4
3. Temporal patterns of flying fox roost formation 5
4. Characteristics of active roosts over time 5
5. Change in flying fox populations in southeast Queensland (SEQ) 6
6. Monthly variations in climate 6
7. Periods of acute food shortages 6
    - Supplementary Table 1 8
    - Supplementary Table 2 13
    - Supplementary Table 3 14
8. Relationship between foraging sites and roost sites 14
    - Supplementary Table 4 15
9. Characteristics of foraging areas over time 16
10. Identify the source roost of spillover 17
11. Pulses of productive winter flowering 17
    - Supplementary Table 5A-B 18
12. Change in winter habitat in far southeast Queensland (SEQ) 1996-2018 19
13. Multiscale Bayesian Network Model 21
    - Supplementary Figure 1 23
    - Supplementary Table 6 25
    - Supplementary Table 7 27
    - Supplementary Table 8 28
    - Supplementary Table 9 29
14. References 32

Datasets

We used the following datasets in the analyses described herein; all datasets are described and summarized in the Data Index[7]. Those generated for the study are either stored in the eCommons open access digital repository (Datasets A, B, C, E, F, G, I, J) and can be accessed via DOIs provided in the reference list at the end of this document, or are provided in a Supplementary Table (Dataset H). Those we compiled from existing public online repositories (Datasets D, K, L, M) can be accessed via their listings in the Data Index.

Data Index: Overview of study datasets, structure, sources, availability, associated analyses[7]

Dataset A: Register of Hendra virus spillovers to horses[12]

Dataset B: Register of flying fox roosts in the study area[26]

Dataset C: SEQ monthly roost distribution and population estimates[27]

Dataset D: Oceanic Niño Index (ONI)[7]

Dataset E: Months of nectar shortage[29]

Dataset F: Records of wildlife rehabilitation centers[30]

Dataset G: Assessments of pre-weaning reproductive output[31]

Dataset H: Paired roost foraging data - Supplementary Table 4

Dataset I: Landcover data[28]

Dataset J: Winter flower pulses[32]

Dataset K: Modelled pre-clearing vegetation types, Southeast Queensland bioregion[7]

Dataset L: Forest cover[7]

Dataset M: Queensland Statewide landcover and trees study[7]

Food Shortage Regression Tree Model; compiled input data, output data and model script[33]

Bayesian Network Model; compiled input data and output figure[34]

1. Register of Hendra virus spillover events in Australia

Data on the location and date of all detected Hendra virus spillovers to horses from its initial detection in 1994 until July 2021 were collated from government notices (New South Wales Department of Primary Industries; Biosecurity Queensland, Queensland Department of Agriculture and Fisheries; Business Queensland), ProMED-mail and local media reports, and personal communications during spillover events (Data Index[7], Dataset A[12]). As of 31 January 2021, a total of 63 Hendra virus spillover events had been detected in Australia. The locations of infected properties (IP) are reported as the nearest town or regional center. Dates represent known or estimated dates of horse death or euthanasia. The putative date of spillover was calculated as 14 days prior to date of death or euthanasia of the first horse infected on a property, based on epidemiological observations of the incubation period in horses[48].

The probability of detecting Hendra virus spillovers may have varied over time. Around midwinter 2008, the Hendra virus case definition in horses broadened to include a febrile horse exhibiting respiratory or neurological signs, leading to an increase in testing[49]. At the same time, incorporation of a new molecular assay into screening may have increased sensitivity of detection[9]. Nevertheless, we did not observe an increase in spillover events post-2008 until after the 2010 food shortage. In November 2012, an equine vaccination for Hendra virus was released[50], likely reducing the incidence of spillover. Vaccination should decrease the number of observed spillover events but vaccination rates are relatively low[51]. We do not expect these factors have affected the overall pattern of observed events reported in this study (ONI→food shortage→Hendra virus spillover).

A novel Hendra virus variant, genotype 2 (HeV-g2), was discovered retrospectively in 2021 in a horse that died after acute illness in 2015[52]. Importantly, HeV-g2 is not detected by the diagnostic assays used to detect prototype HeV (HeV-g1) in our study area prior to 2021 due to the high specificity of assays and the 84% pairwise nucleotide identity genome-wide between the two variants[52]. The variant could not have been detected in Hendra virus surveillance efforts in our study area from 1994 until 2021 and therefore does not affect the results of this study. HeV-g2 RNA has been detected in flying fox tissue samples[53] and in flying fox urine[54], however in the absence of data on rates of HeV-g2 spillover to horses prior to 2021, we have focused our analyses on HeV-g1 spillover. Further data on spillover of both variants over coming years will be required to assess whether results from our analyses of HeV-g1 (hereafter, Hendra virus) also apply to HeV-g2.

2. Study area

Our study was conducted in the subtropics of eastern Australia within an area where 65% of all Hendra virus spillovers recorded from 1994 to 2020 were located (41 of 63, Dataset A[12]). To define the boundary of our study area, we placed 100 km buffers around subtropical spillover sites recorded in 2011, a year when a uniquely high number of spillovers were recorded[12] (n=17) and constructed a minimum bounding shape around the perimeter (Fig. 1, Extended Data Fig. 1A). Data collection and collation for our study was focused within this area. Two spillovers that occurred in the subtropics outside of this study area were omitted (Calliope, Queensland, 2014; Scone, New South Wales, 2019).

Detailed surveys of flying fox roosts in the far southeast region of Queensland, commencing in 1996, defined a smaller southeast Queensland (SEQ) study area within the larger study area (Extended Data Fig. 1B, Dataset B[26], Dataset C[27]). The boundary of the SEQ study area was defined

by placing buffers scaled to 20 km feeding distances[55] around the 1996 flying fox survey sites and dissolving all buffers to a single area.

Hereafter, 'study area' refers to the larger study area based on spillover detection, and 'SEQ study area' is used to refer to the smaller study area that focused on flying fox population dynamics and loss of foraging habitat.

3. Temporal patterns of flying fox roost formation

To describe long-term patterns of spatial ecology and roost formation, we created a register of active roost sites within the study area, compiled over a 25-year period from 1996 to 2020 (n = 361 roost sites; Dataset B[26]). We acquired information from multiple sources: records held by New South Wales (NSW) and Queensland (Qld) state governments; broad-scale surveys conducted 1998–2005 and 2012-2020; records held by local land managers, landowners, experienced observers and field records of authors (see Data Index[7] for further details and references). Data recorded in the register include roost name, location (latitude, longitude), state, year of formation, the most recent year of recorded occupation, occupation in winter and occupation by black flying foxes *Pteropus alecto* in winter of each year.

'Active roosts' were defined as roosts known to be occupied in the previous 10 years, which is consistent with the timeframe used by the Australian Commonwealth for identifying 'nationally-important camps'[56]. Year of roost formation was allocated based on the date of first observation by landowners, land managers and neighbors[7]. Roosts that were established prior to the start of our ecological study-period in 1996 were assigned 1996 as their establishment date, although many had longer histories of occupation. Where year of first observation was uncertain, indicative categories were assigned. These sites were excluded from assessments based on year of formation. Overall, we were able to assign year of formation to 88% of the roosts formed after 1996.

4. Characteristics of active roosts over time

We used year of formation and year of most recent observation to compile lists of active roosts for each of the 25 years in our longitudinal study (Supplementary Information Section 3, Dataset B[26]). Then for each active roost in each year we assembled data on 1) the mean distance to nearest active roosts, 2) binary occupation by flying foxes during winter months, 3) binary occupation by black flying foxes *Pteropus alecto* during winter months, and 4) identification of the site as the source roost for spillover(s). The mean distance to nearest active roosts was calculated from coordinates of the target roost and the nearest active roost in quadrats defined by cardinal directions. Source roosts for Hendra virus spillover were assumed to be the nearest roost to the spillover based on analyses presented in Supplementary Information Section 13.

Records of winter occupation were collated from the sources for Dataset B listed in the Data index[7]. Range-wide winter censuses of flying foxes commenced in the study area in 1998[17] and few roosts were directly observed in winters of 1996 and 1997. Unobserved roosts were initially assigned 'NA' status. Expert knowledge and census data from the years that followed were then used to assign presumptive winter occupation status as follows.

1) Roosts located outside the known range of black flying foxes were presumed unoccupied by black flying foxes. A southward shift in the range of that species was documented during our study; those data guided assignment of roost occupancy[57].

2) Expert knowledge and census data from adjoining years or near adjoining years (1998-2002) were used to assign occupancy to roosts where patterns were consistent over 2-3 consecutive years.

3) 'NA' status was assigned to all other roosts.

5. Change in flying fox populations in southeast Queensland (SEQ)

To describe change through time in the distribution of flying foxes in the far southeast Queensland region, we compared monthly population estimates of black flying foxes and grey-headed flying foxes (*P. poliocephalus*) recorded in roosts during two time periods: 1996-1999 and 2009-2012 (Dataset C[27]). Roosts that formed within the SEQ study area boundary were added to surveys as they were reported. A total of 33 monthly population surveys (synchronized across all roosts so that they occurred within 3 consecutive days) were conducted in 1996–1999; 39 surveys were conducted in 2009–2012. These data provided information on the number of roosts, population estimates of each species at individual roosts, and the total estimated population size within the study area.

The total number of flying foxes present in the study area within each time period was variable due to the high mobility and irregular migration patterns typical of the species[14,15,22,58]. The data describe monthly aggregated change in roost size between the two study periods, a decline in combined species estimates and consistency in the seasonal estimates of black flying foxes (Extended Data Fig. 4A-C).

6. Monthly variations in climate

Broad-scale variations in climate were tracked using monthly readings of the Oceanic Niño Index (ONI; Fig. 2A, see Dataset D, see Data Index[7]). The ONI was developed by the USA National Oceanic and Atmospheric Administration to monitor and rank the relative strength of the El Niño-Southern Oscillation. It tracks anomalies in running 3-month average sea surface temperatures in the east-central tropical Pacific Ocean between 120° and 170°W (the Niño 3.4 region) against 30-year averages to produce the ONI value for a 3-month interval. Values ≥ 0.5 are classified as El Niño, values <-0.5 are classified as La Niña.

We compiled monthly ONI readings from December-January-February 1996 to November-December-January 2020 (Fig. 2A).

7. Periods of acute food shortages

Flying foxes in southeast Australia have been reported to experience periods of reduced nectar production in native diet plants that impact fitness via reduced nutrient intake and starvation[22,59]. We sought to test the association between the presence or absence of nectar shortage in flying fox diet species (as assessed by qualitative reports of nectar productivity from commercial apiarists) and

anomalies in measures of flying fox fitness using a regression tree analysis. Additionally, since nectar productivity data is a commercial resource for apiarists and, therefore, difficult to obtain, we sought to test whether more readily obtainable measures of flying fox fitness were a useful proxy for acute food shortages.

7.1 Periods of Nectar Shortage - Background and Methods

BACKGROUND

The commercial apiary industry in southeast Australia comprises enterprises with ≥50 hives who respond to spatio-temporal variations in nectar production by moving their hives to maximise honey production and minimise costs of supplemental feeding[60]. Commercial apiarists primarily utilize native forests and adopt nomadic practices to track highly variable nectar and pollen resources[61], much like nomadic bats. The tree species utilized by the *Pteropus* bats in southeast Australia are also utilized by commercial apiarists (Supplementary Table 1) and European honeybees in the region experience the same winter/spring periods of food bottleneck as do nectar dependent bats[62]. In the absence of direct and extensive data on nectar availability we have used data derived from commercial apiarists to provide a spatio-temporal response variable of nectar availability that is produced independently to the flying fox study presented here. This variable is based on the deep system knowledge and multi-generational experience of apiarists[61,62]; however, it is a subjective assessment and as such, we determined the simplest and most accurate response should be a binary response recording either a nectar shortage or not.

Extensive nectar monitoring: The development of detailed and quantitative methods to systematically track eucalypt flowering using remote sensing techniques is an area of active research, funded to support the apiary industry[63]. However, progress has been slow, and an accurate and comprehensive method is unlikely in the near future[64]. For several years we have used the commercial records of individual apiarists to track these resources. Initially, we used this approach to characterize flowering pulses associated with the movements of individually radio-tagged *Pteropus poliocephalus*[14]. Subsequently this was extended to identify periods of broad-scale food shortage and predict flowering after periods of food shortage[24].

METHODS

We aim to produce a binary, monthly assessment of periods of absence or near absence of nectar production from bat diet plants in native forests in southeast Australia. We have identified that the most consistent method for spatio-temporal assessment of nectar shortages is through direct communications with the commercial apiary industry and our method is based on verbal communications with individual apiarists and their representatives. Information on the availability of native food resources is highly valued during commercially competitive periods of nectar shortage and the acquisition of accurate, commercial reports from apiarists is typically provided in confidence.

Network of contacts: We established a network of contacts comprising a core group of 1) apiarists that manage substantial enterprises (hundreds of hives), 2) active members of industry support groups (federal, state and local industry associations, government advisory personnel) and 3) honey packers to whom apiarists sell their products. Members in each of these categories track nectar resources over substantial areas either directly or through their member groups and other connections.

Surveys: We communicated with members of our contact group verbally (via telephone) which enabled us to seek additional information or clarification at the point of contact if needed while at the same time avoiding sensitivities over the confidentiality of written responses.

Supplementary Table 1. Overlap between nectar sources for bats and bees assessed by characterizing native species in the diet of *Pteropus* species[16] using scores of the importance of native plants as resources for the apiary industry[62]. Species are scored on a scale of 1 to 5 against a set of standard criteria that consider the nutritional value of nectar and pollen, distribution, and hive management including disease management. Of the 53 native trees on the bat diet list, 93% (49) were assigned scores of importance to the apiary industry of 3 or higher, median score = 3. Four of the seven species that contributed to winter flower pulses in this study, highlighted in grey, (Extended Data Fig. 9) were assigned the highest score of 5.

Species	Common name	Apiary score scale 1-5	Comments
Corymbia maculata	Spotted Gum	5	
C. variegata	Northern Spotted Gum	5	
Eucalyptus albens	White Box	5	
E. camaldulensis	River Red Gum	5	
E. macrorhyncha	Red Stringybark	5	
E. melliodora	Yellow Box	5	
E. muelleriana	Yellow Stringybark	5	
E. paniculata	Grey Ironbark	5	
E. siderophloia	Grey Ironbark	5	
E. sideroxylon	Mugga Ironbark	5	
Lophostemon confertus	Brush box	5	
Melaleuca. quinquenervia	Five-veined Paperbark	5	
C. gummifera	Red Bloodwood	4	
E. andrewsii	New England Blackbutt	4	
E. fibrosa	Broad-leaved Ironbark	4	
E. pilularis	Blackbutt	4	
E. saligna	Sydney Blue Gum	4	
E. acmenoides	White Mahogany	4	
Angophora costata	Smooth-barked Apple	3	
A. floribunda	Rough-barked Apple	3	
Banksia serrata	Old Man Banksia	3	
B. integrifolia v. integrifolia	Coast Banksia	3	
C. eximia	Yellow Bloodwood	3	
C. henryi	Large-leaved Spotted Gum	3	
C. intermedia	Pink Bloodwood	3	
C. trachyphloia	Brown Bloodwood	3	
C. citriodora citriodora	Lemon-scented Gum	3	
E. campanulata	New England Blackbutt	3	
E. deanei	Mtn Blue Gum	3	
E. grandis	Flooded Gum	3	
E. maidenii	Maiden's Gum	3	
E. melanophloia	Silver-leaved Ironbark	3	
E. parramattensis	Parramatta Red Gum	3	
E. piperita	Sydney Peppermint	3	
E. planchoniana	Needlebark	3	
E. punctata	Large-fruited Grey Gum	3	
E. resinifera	Red Mahogany	3	
E. rummeryi	Steel Box	3	
E. seeana	Narrow-leaved Red Gum	3	
E. tereticornis	Forest Red Gum	3	
E. tricarpa	Red Ironbark	3	
Syncarpia glomulifera	Turpentine	3	
C. tessellaris	Carbeen	2	
E. amplifolia	Cabbage Gum	2	
E. botryoides	Southern Mahogany	2	
E. moluccana	Grey Box	2	associated with fermentation of honey in hives
E. propinqua	Small-fruited Grey Gum	2	
E. robusta	Swamp Mahogany	2	identified as source of *Nosema ceranae* infections
Grevillea robusta	Silky Oak	2	highly restricted distribution
E. bancrofti	Orange Gum	no score	sparsely distributed
E. cloeziana	Gympie Messmate	no score	distribution outside area considered
E. major	Grey Gum	no score	restricted distribution
Castanospermum australe	Black bean	no score	sparsely distributed

We requested assessments of the native tree species apiarists 'worked' (targeted nectar source) at monthly intervals, the locations where their hives were placed, a qualitative indication of productivity of the resource and, where possible, an indication of the anticipated duration of the resource. Apiarists also monitored budding in order to anticipate and plan movement of hives in coming months and we elicited information about this when available. We verified reports using one or more independent members of our network.

We used our knowledge of the native diet plants used by bats (Supplementary Table 1) and their distribution[16] to target efforts. We commenced a monitoring period by eliciting information on resources in the most consistently productive regions in our study area: southeast Queensland and northeast New South Wales. If we were able to verify productive flowering from diet plants in this region, we classified the month as "not a food shortage". If we were unable to verify productive flowering, we expanded the area of surveillance to include other potentially productive regions.

In our experience, when there was a surplus of nectar in native forests, our contacts were open and forthcoming with information. When conditions were marginal and there was competition for information on productive forest areas, greater efforts were required to verify conditions. When acute conditions occurred, we expanded the range of the apiarists we contacted to capture more detailed local knowledge. At that point our contacts reported the changes they made to management practices to mitigate the impact of nutritional stress and risk of starvation to their bees. Those practices included withdrawing hives from native forests, supplementary feeding, movement of hives onto crops for which no financial reward was received and sacrificing hives to maintain a core, healthy group.

If we were unable to verify nectar production in native forests or were made aware of starvation management practices, we classified the month as "nectar shortage".

Data were compiled over a 22-year period from January 1998 to February 2020 (Dataset E[29]). We recognise that this method of data acquisition is subjective and time consuming. The regression tree analysis presented in this manuscript represents an initial endeavour to develop a proxy for food shortage based on reliable, long-term data that sit outside the constraints of one-on-one commercial in-confidence experiences. We are now developing this by using explainable AI techniques to develop accurate decision trees for identifying food shortage months based on environmental and ecological variables.

The Griffith University Human Research Ethics Committee approved the research (GU Ref No: 2022/765) and informed consent was obtained from all human research participants.

7.2 Wildlife rehabilitation intake data

We identified three measures of fitness in flying foxes for which long-term data were available in our study area: rates of intake into wildlife rehabilitation centers, body mass recorded by animal rehabilitation centers at the time of encounter, and annual rates of pre-weaning reproductive output (Dataset F[30], Dataset G[31]). Flying foxes present to wildlife rehabilitators due to poor condition, illness or injury. We assume that in the absence of treatment, rescued flying foxes would likely die, and therefore use data on flying fox intakes as a proxy for mortality. We used the records of wildlife rehabilitation organizations in the Hendra virus study area to track trends through time in intake and body mass (g) of black flying fox *P. alecto* (BFF), grey-headed flying fox *P. poliocephalus* (GHFF), and little red flying fox *P. scapulatus* (LRFF) (Dataset F[30]). Data were compiled over nearly 20-years from January 1998 – May 2003 and January 2006 – February 2020 in the Northern Rivers (NR) region of NSW (latitude -28.5 to -29.0). We focused on this region because it is located near the

latitudinal midpoint of our study area, 11 spillovers were recorded within its boundary from 1994 to 2020 and because wildlife rehabilitation data was recorded consistently using standard methods over the extended data collection period. Trends in these data are expected to be broadly representative across the remainder of our study area.

Encounter records: We acquired daily records of flying foxes encountered by rehabilitation practitioners, initially from the Northern Rivers Wildlife Carers rehabilitation group, and from the NR Branch of the Wildlife Information Rescue and Education Service (NR WIRES) commencing in 2006, the year that branch formed (Dataset F[30]). No records were available from June 2003 through December 2005. Data were filtered to exclude short spikes (1-2 days) in encounters associated primarily with impacts of severe weather conditions, notably extreme heat identified by days when maximum ambient temperature exceeded 42°C[65].

Morphometric data: Body mass (g) and forearm length (mm) of adults were included in the data if the measures were taken prior to treatments such as rehydration that could influence body mass (Dataset F[30]). These measurements were not routinely collected, limiting their power within analyses. From January 2006 – February 2020, pre-treatment body mass records faceted to age, species and sex were uncommon and highly inconsistent, particularly in non-food shortage periods when few individuals were handled. Data collated from 1998-2003 included fewer pre-treatment mass and forearm measures and this time period was therefore not included in the regression tree analysis.

7.3 Annual reproductive output

Poor nutrition during winter/spring food shortages coincides with late gestation, birth and early lactation in adult females and is expected to affect annual reproductive success via loss of pregnancy or insufficient lactation leading to pup starvation. We assessed annual reproductive output at roosts using the percentage of females carrying young in late December/early January (pre-weaning) (Dataset G[31], Extended Data Fig. 2A). A minimum of 10 roost trees containing adult reproductive groups was selected randomly in each roost. In each tree, an individual animal was selected and, using binoculars, the sex and reproductive status of nearest neighbors were recorded until 10 adult females had been sampled ($\geq$ 100 females sampled per roost per year). *P. alecto* and *P. poliocephalus* conception occurs in April, births center around October, and weaning occurs immediately prior to the subsequent mating season. Measurements of pre-weaning reproductive output from January were therefore applied in the model to the output from that entire birth cohort (months of April through March).

Assessments of pre-weaning reproductive output were made at a total of 15 roosts in our study area during the 23-year study, and the roosts sampled varied between years due to variations in accessibility, occupation and the number of adult females present (Dataset G[31]). Sites affected by extreme temperature in the weeks preceding annual assessments were excluded. To be spatially consistent with the wildlife rehabilitation intake and body mass data, reproductive output data for input in the model was acquired from 4 roosts in the NR region (Booyong, Lumley Park, Rotary Park and Kyogle roosts).

7.4 Oceanic Niño Index (ONI) Data

Finally, we incorporated monthly Oceanic Niño Index (ONI) measurements from National Oceanic and Atmospheric Administration as an index of broad-scale variations in climate (Supplementary Information Section 3, Dataset D[7]).

7.5 Regression Tree Methods

The regression tree approach aims to identify the simplest set of rules that can be used to assign known classes according to potential explanatory variables. Here it is used to first assess whether various explanatory variables (including ONI, flying fox encounter records, pre-treatment body mass, and pre-weaning reproductive output) can correctly identify months of presence or absence of nectar shortage as recorded from apiary data (and thereby demonstrate their potential as proxies). The Recursive Partitioning algorithm 'rpart'[66] splits the data recursively into groups, so that at each stage, the partition is made based on the explanatory variable that results in increasingly homogenous groups with respect to the presence or absence of flowering. Resulting models can be represented as binary decision trees. All data not already a monthly measurement was aggregated or divided to the month level and was restricted to the period for which consistent data were available: the 14-years between January 2006 – February 2020[33]. The regression tree was fit from the 'rpart()' function in the 'rpart' R package, that results from minimizing 10-fold cross validation error[66].

7.6 Features assessed in the food shortage predictive model

1. NUMBER OF FLYING FOXES ENCOUNTERED BY NR WIRES
    - Monthly count of flying foxes in the records of NR WIRES,
    - One-month lag of monthly counts.
2. MORPHOMETRIC DATA
    - Adult BFF female mass (minimum monthly, lower 0.25 quantile, mean)
    - Adult BFF male mass (minimum monthly, lower 0.25 quantile, mean)
    - Adult BFF female mass/forearm (minimum monthly, lower 0.25 quantile, mean)
    - Adult BFF male mass/forearm (minimum monthly, lower 0.25 quantile, mean)
    - Adult GHFF female mass (minimum monthly, lower 0.25 quantile, mean)
    - Adult GHFF male mass (minimum monthly, lower 0.25 quantile, mean)
    - Adult GHFF female mass/forearm (minimum monthly, lower 0.25 quantile, mean)
    - Adult GHFF male mass/forearm (minimum monthly, lower 0.25 quantile, mean)
3. ANNUAL REPRODUCTIVE OUTPUT
    - Annual Dec/Jan measurements of percent adult females with young applied to months of April through March (for Booyong/Lumley roost, for Rotary Park/Kyogle roosts, and for mean and minimum across both roosts)
4. ONI
    - Indicator if the month is Sep/Oct/Nov/Dec and if an ONI of greater than or equal to 0.8 occurred within last 11 months.

7.7 Results

The apiarist data identified 9 years in which periods of nectar shortage occurred, all within winter or spring (1998, 2000, 2003, 2007, 2010, 2012, 2013, 2016, and 2019). They ranged in length from 1 to 6 months (Supplementary Table 2, Dataset E[29]).

Using data collected from 2006 to 2009, the regression tree selected two explanatory variables for identifying months of reduced fitness (referred to here as food shortage): monthly counts of flying foxes recorded by NR WIRES and minimum percent females with young in local roosts the following January[30,31] (Extended Data Fig. 3). Each of these measures varied widely during the 2006-2020 study

period: monthly counts of flying fox intakes ranged from 0 to 150 (median 14, n = 170) and minimum percentage females with young ranged from 3 to 99 (median 87, n = 37).

All months with counts of flying foxes greater than or equal to 30, and with a minimum percentage of females with young pre-weaning of less than 79, were classified by the model as a food shortage month, while all other months were classified as non-food shortage[33] (Extended Data Figs. 2 A, B, 3). On a monthly basis, using the two classification rules, the model was highly accurate: 164 out of 170 months (96.5%) were consistent with the apiarist classifications (Supplementary Table 2,3). On an annual basis, the model identified five of the six apiarist identified nectar shortage years between 2006 and 2019: winter and/or spring months of 2007, 2010, 2013, 2016 and 2019, and all 9 of the non-nectar shortage years (Supplementary Tables 2,3). It did not identify the short food shortage identified by apiarists in October 2012 (see more detail below).

A small number of months (n=6) were identified as food shortage by one method but not both methods (Supplementary Table 2). Firstly, three months (in 2013 and 2019) were identified as shortages by the model, but not by apiarists. Each of these three months immediately preceded or immediately followed months of shortage identified by both apiarists and the model and contained a small number of days at the beginning or end of the period of rapid increase in encounters. The influence of those days was sufficient to push encounter numbers over the classification threshold (≥30) but was not sufficient for apiarists to classify the entire month as nectar shortage. Secondly, two months (August and September in 2010) out of the three identified as shortages by apiarists but not by the model, presented as dips in the monthly flying fox intake in the middle of an extended and severe 6-month food shortage, with correctly assigned months on either side (July/August and October/November). None of these mismatches affected the binary designation of a presence or absence of a food shortage in those years. Finally, the remaining month that was not identified as a food shortage month by both methods, October 2012, was identified as a nectar shortage by apiarists, but not as food shortage by the model. It was the only month in 2012 that was identified by either method and was the only instance where the model failed to predict a year identified as nectar shortage by apiarists.

When applying the classification rules from the regression model to intake data and reproductive output data available from 1998–2003, three additional years were identified as food shortages. All three years (1998, 2000 and 2003) were consistent with apiarist data and 60 out of 60 months from 1998–2002 were correctly assigned. From 2003–2005, reproductive output data could be used to assign presence/absence of a food shortage year, however the absence of monthly intake data precluded resolution of the result to months (Supplementary Table 2, Dataset F[30]).

To further explore the relationship between the nectar shortage and bat fitness data, we also applied the model output to fitness data acquired in the Mid North Coast (MNC) region of NSW. The MNC WIRES branch is located approximately 90 km south of the southern extent of the NR region. Flying fox count data were acquired from the MNC WIRES rehabilitation center over 183 months from 2005-2020 (Dataset F[30]) and reproduction data was acquired from 3 roosts in the region (Bellingen Island, Nambucca Heads and Bowraville, Dataset G[31]). The distribution of the data was consistent with data from the NR: monthly flying fox counts ranged from 0 to 140, median 6; pre-weaning reproductive output ranged from 4 to 99, median 86.

Supplementary Table 2 – Summary of months in each year between 2006–2020 assessed as food shortages in apiarist data and by the regression tree model (Northern Rivers)

Year	**Food shortage months identified by apiarists**	**Food shortage months identified by regression tree (2006-2020) or regression tree rules (1998-2003)**	**Number of months consistent across methods**	**Comments**
1998	Oct, Nov	Oct, Nov	12	
1999	-	-	12	
2000	Sep, Oct, Nov	Sep, Oct, Nov	12	
2001	-	-	12	
2002	-	-	12	
2003	Jul, Aug	2003 *	*	*Partial data
2004	-	- *	*	*Partial data
2005	-	- *	*	*Partial data
2006	-	-	12	
2007	Jul, Aug, Sep, Oct, Nov	Jul, Aug, Sep, Oct, Nov	12	
2008	-	-	12	
2009	-	-	12	
2010	Jun, Jul, Aug, Sep, Oct, Nov	Jun, Jul, Oct, Nov	10	Intake in Aug (n = 26) and Sep (n = 15) was below threshold.
2011	-	-	12	
2012	Oct	-	11	Reproductive output data was consistent with food shortage in nearby Mid North Coast roosts (min 63) but not at the single roost assessed in the Northern Rivers
2013	Nov	Oct, Nov	11	Daily intake data indicate that flying fox encounters started to increase on 26th October, enough to push October intakes over the threshold (n = 32).
2014	-	-	12	
2015	-	-	12	
2016	Nov	Nov	12	
2017	-	-	12	
2018	-	-	12	
2019	Sep, Oct, Nov	Aug, Sep, Oct, Nov, Dec	10	Daily intake data indicate that flying fox encounters started to increase on 22nd August, pushing August intakes over the threshold (n = 42). Encounters were elevated until 9th December, also pushing December intakes over the threshold (n = 44).
2020	-	-	12	

* Partial Data: Reproductive output is consistent with a food shortage in 2003, but no winter/spring encounter data is available to enable predictions of months. Reproductive output is consistent with no food shortage in 2004 or 2005, but there is no encounter data to fully assess.

Supplementary Table 3. Summary of months between 2006–2020 assessed as food shortages by apiarist data and by the regression tree model (Northern Rivers)

	Model: shortage	Model: not shortage	Total	% model output consistent with apiarist data
Apiarist: shortage	**14**	3	17	82.4%
Apiarist: not shortage	3	**150**	153	98.0%
Total	17	153	**170**	96.5%

When we applied the rules from the regression tree model to these data to classify months as food shortage (monthly flying fox encounters ≥30 and pre-weaning percentage females with young <79), the annual designations classified consistently with the apiarist assessments in all 17 years and the monthly designations classified consistently with the apiarist assessments in 173/183 (94.5%) of the months. The food shortage in October 2012 was clearly identified as such, based on 68 encounters and minimum 63 percent reproduction.

Our two aims in these analyses were to (1) test the association between data on nectar productivity collected from apiarists (as a proxy for nectar availability for flying foxes) and measures of flying fox fitness, and in turn, (2) assess whether fitness measures are a useful proxy for identifying years of acute food shortages. Given that nectar shortage conditions identified by apiarists were correctly identified by the regression tree model in 96.5% of months over a 14-year period using two variables of flying fox fitness in the Northern Rivers data (flying fox intakes to wildlife rehabilitators and annual reproductive output), and in 94.5% of months over a 15 year period in the Mid North Coast data, our findings support the expectation that reduced flowering in native diet plants affects flying fox survival and reproductive output, likely via starvation.

Overall, the strong correlation between the apiarist assessment of food shortages and the monthly number of flying foxes recorded by wildlife rehabilitation organizations and percent of females with young in the associated birth cohort indicates that either of these approaches are likely to be suitable proxies for food shortages in flying fox populations. We note that reproduction data should be gathered from multiple roosts to accommodate the underlying variability in food shortage years shown in Extended Data Figure 2A. On the basis of these results and the consistent acquisition of apiary data throughout the study period, we used apiarist data to assign years of food shortages in the Bayesian Network model (Supplementary Information Section 13) and months of food shortages in Fig. 2 and Extended Data Fig. 2B. The 9 years of acute food shortage identified in our study area were: 1998, 2000, 2003, 2007, 2010, 2012, 2013, 2016 and 2019. We also conclude that future studies could reasonably use flying fox intakes and reproductive output to identify food shortage years where apiarist data is unavailable or prohibitively difficult to acquire.

8. Relationship between foraging sites and roost sites

Foraging areas around flying fox roosts in southeast Australia have previously been defined by buffers of 20km radius, an area understood to encompass the typical foraging distances of the animals[55]. This approach assumes overlap in the foraging areas of neighboring roosts is unconstrained. However, there is increasing evidence that colonial species, including fruit bats, spatially partition the resources around neighboring colonies, particularly those that are separated by distances shorter than typical foraging distances[67,68]. In our study area, rapidly increasing

numbers of roosts are separated by distances considerably shorter than 20km (Extended Data Fig. 5A) and we propose that a set radius of that length no longer adequately describes the foraging areas of the majority of roosts. Rather, we propose that as the number of roosts in a local area increases, flying foxes reduce the distances they fly between roosts and foraging sites (individual feeding trees) and typically occupy the roost closest to their foraging sites.

8.1 Methods

To test this concept, we assessed the relationship between foraging sites and roosting sites in black flying foxes (BFF; *P. alecto*) and grey-headed flying foxes (GHFF; *P. poliocephalus*) using foraging movement data compiled from ten telemetry studies (n=6 studies of BFF, n=4 studies of GHFF) conducted between 1989 and 2015 (Data Index[7] Dataset H, Supplementary Table 4).

Studies were selected for inclusion if:

1. presence / absence occupancy status of neighboring roosts was known at the time of the study; and
2. the locations of roosts and foraging sites were accurately documented.

Roosting site-foraging site pairs (R-F pairs) were included if:

1. the study animal departed from and returned to the same roost during the foraging bout, creating an unambiguous association between roost site and foraging site;
2. the nearest neighboring roost was < 20km distant;
3. the pairwise association described a discrete R-F pair (i.e. eliminating replication from repetition in nightly foraging bouts.)

Supplementary Table 4. Records of 251 paired roosting sites and foraging areas (R-F pairs) of grey-headed flying foxes (GHFF; *P. poliocephalus*) and black flying foxes (BFF; *P. alecto*) as documented in 10 telemetry studies conducted from 1989 to 2015. Counts and proportions of R-F pairs where study animals occupied the roost nearest their foraging sites are given, summarized by species, telemetry type and source study. Data sources are fully referenced in Data Index[7] Dataset H. The data indicated that BFF and GHFF occupy the roost located nearest their foraging sites 95% (n=63) and 96% (n=188) of the time, respectively.

Study location and years	Telemetry Type	Dominantland cover type(s) in study area	Species	Number of R-F pairs	Number R-F pairs & nearest roost	Proportion R-F pairs & nearest roost
Sunshine Coast, QLD; 2015	Satellite	urban, agriculture, forest	BFF	4	4	1
RBGS; Sydney NSW; 2011	Radio VHS	Urban	BFF	6	5	0.83
RBGS; Sydney NSW; 2012	Radio VHS	Urban	BFF	15	15	1
Roberts; Maclean NSW 2012	Satellite	urban, agriculture, forest	BFF	10	10	1
Markus; Brisbane QLD 1998-2000	Radio VHS	Urban	BFF	16	15	0.94
RBGS; Sydney NSW; 2010	Radio VHS	Urban	BFF	12	11	0.92
Eby; NE NSW; 1989-1990	Radio VHS	agriculture, forest	GHFF	12	12	1
RBGS; Sydney NSW; 2010	Satellite	urban, agriculture, forest	GHFF	12	9	0.75
RBGS; Sydney NSW; 2011	Radio VHS	Urban	GHFF	13	13	1
RBGS; Sydney NSW; 2012	Radio VHS	urban, forest	GHFF	18	17	0.94
Roberts; Sth QLD NSW; 2007-2008	Satellite	urban, agriculture, forest	GHFF	133	130	0.98

For each species we calculated the proportion of R-F pairs in which study animals occupied the roost nearest the foraging site. To obtain intervals expressing uncertainty in the estimates, a hierarchical bootstrap was performed with 10,000 bootstrap samples, combining data across sources within a species. For GHFF, an additional analysis was performed using data from one study that provided the majority of data points (Supplementary Table 4; Roberts 2007-2008). The results of the hierarchical bootstrap were compared with the regular bootstrap procedure, and the more conservative estimates used.

8.2 Results

The dataset comprised 251 R-F pairs. The data indicated that BFF and GHFF occupy the roost located nearest their foraging sites 95.2% (n=63) and 96.3% (n=188) of the time, respectively. The proportion of flying foxes returning to the nearest roost after foraging did not differ meaningfully by study (Supplementary Table 4, and below). Bootstrapping produced the following 95% confidence intervals (95% CI):

All BFF (6 studies):
n = 60 return to nearest roost from n=63 data points; 95.2%
95% CI: 0.889–1.000.

All GHFF (4 studies):
n = 181 return to nearest roost from n=188 data points; 96.3%
95% CI: 0.931–0.989

GHFF Roberts (2007-2008) only:
n = 130 return to nearest roost from n=133 data points; 97.7%
95% CI: 0.947 –1.000

Based on these results, we assume BFF and GHFF occupy the roost nearest their foraging site. To define foraging area boundaries, we set a maximum foraging distance of 20km, then constrain this boundary by the mid-point of distances to neighboring roosts, which we define by Voronoi cells. We believe this to better approximate foraging area boundaries than uniform radius and we used this method to define and characterize foraging areas associated with individual roosts (Supplementary Information Section 9) and identify source roosts for Hendra virus spillovers (Supplementary Information Section 10).

9. Characteristics of foraging areas over time

We described long-term change in characteristics of the foraging areas available to flying foxes in the study area by comparing a series of attributes of the foraging areas associated with roosts active in 1998 and 2019 (Dataset B[26]; Supplementary Information Section 4).

Based on the identified relationship between foraging sites and roost sites (Supplementary Information Section 8), we defined the boundaries of discrete foraging areas around individual roosts by calculating Voronoi tessellations with roost sites as point locations and 20 km foraging distances[55] as the window boundary ('Spatstat' package in R[69]). Voronoi diagrams of the study area were recalculated in each year based on the list of active roosts for that year. We considered that roosts known to have been vacant during the previous 2 years did not influence the foraging areas of neighboring sites, and those roosts were excluded from calculations. Census data and informal observations of roosts were scrutinized to determine recent occupation.

To describe the increase in roosts in urban areas we plotted the scaled density of distance to nearest built land (Extended Data Fig. 5B). To summarize changes in the relationship between foraging area size and the proportion of the foraging area that is built land we fit a generalized additive model (GAM) with a thin plate regression spline smoother using the 'mgcv' package in R[70]. We used foraging area size as the predictor and proportion of the foraging area that is urban as the response and plotted the smoothed regression line (Extended Data Fig. 5C).

We then characterized changes in the land cover composition of foraging areas associated with roosts occupied by black flying foxes during winter in each of the 25 years of our study, 1996 through 2020 (Dataset B[26]). We created boundaries for the foraging areas of roosts occupied in each winter using the methods described in Section 8. We used historical 30-m resolution land cover maps created at 5-year time steps[71] to characterize changes in the land cover composition of foraging areas (Dataset I[28]). The maps classified land cover into six broad classes: built land, cropland, grassland, forest, water and other. We matched the Voronoi diagrams for each study year to the closest time-step in the map series (1995, 2000, 2005, 2010, 2015) and clipped the landcover map to foraging area boundaries. We then filtered the data to include only sites occupied by black flying foxes. Spatial data was projected to Australia Albers / GDA94 coordinate reference system.

We characterized changes in the land cover composition of the foraging areas associated with each roost by calculating 1) the size of the foraging area (km^2), 2) the area attributed to each of built land, forest and agriculture (combined cropland and grassland), and 3) the proportion of the area attributed to each land cover type. Finally, in each year we calculated the total area of each land classification (built, forest, and agricultural) within the combined foraging areas of the winter roosts of black flying foxes (Extended Data Fig. 5C).

10. Source roost of spillover

Hendra virus transmission occurs as a result of flying foxes foraging on horse properties[8,9]. Using the analyses in Supplementary Information Section 8 that demonstrate that flying foxes roost in sites closest to their foraging areas, we assumed that the occupied roost (Dataset B[26]) closest to the spillover site (Dataset A[12]) was the likely source of bats responsible for the spillover event. We explored the implications of misspecification of the spillover-roost (described in Supplementary Information Section 13.4). For spillovers with a plausible second most likely roost, we explored the impact of shifting the attributions to the second roosts. In total, this resulted in a net shift of one spillover in an urban area to an agricultural area. So even if all the roost assignments were mis-specified, the result would be minimal.

11. Winter flowering pulses

Individual eucalypt species in the diet of flying foxes in the Australian subtropics are productive for periods ranging from 6 weeks to >3 months. The animals are notably adept at tracking these ephemeral resources, and large aggregations of nomadic animals can form rapidly when flowering commences[22]. Few forested areas produce sufficient nectar and pollen to attract roost populations >50,000.

Over 75% of horse deaths from Hendra virus spillovers in the Australian subtropics occur in the Austral winter from June through August (Dataset A[12]; Fig. 1). We observed over 15 years that highly productive, long duration winter flowering of a single species of eucalypt, *Corymbia maculata* spotted gum, coincided with the only years from 2005 to 2020 in which no winter case

Supplementary Table 5A. Evidence of pulses of winter flowering in flying fox diet plants in the subtropics of eastern Australia associated with aggregations of ≥100,000 *P. alecto* and *P. poliocephalus* in a single roost (Dataset J[32]). A key to roosts is provided in Supplementary Table 5B. Data sources are referenced in the Data Index[7].

YEAR	TOTAL SPILLOVERS	WINTER SPILLOVERS	WINTER AGGREGATION	ROOSTS	MONTHS AGGREGATIONS WERE RECORDED
1997	0	0	1	B, E, N	May June July August
1998	0	0	1	F, G	June July August
1999	0	0	-	incomplete data	
2000	0	0	1	E	June July
2001	0	0	1	I, N	May June July August
2002	0	0	1	F, G	June July August
2003	0	0	-	no data	
2004	0	0	-	no data	
2005	0	0	1	N	May June July August
2006	2	1	-	no data	
2007	1	0	0	no aggregation	
2008	1	1	-	incomplete data	
2009	0	0	1	N	May June July August
2010	1	0	1	F	June July
2011	17	16	0	no aggregation	
2012	0	0	1	B, L, M, N	May June July August
2013	6	5	0	no aggregation	
2014	3	1	-	incomplete data	
2015	2	2	0	no aggregation	
2016	1	0	1	B, D, H, O	May June July August
2017	4	3	1	J	May June
2018	1	1	1	A, H	June July August
2019	1	0	1	J, K	June July August
2020	1	0	1	C	July August

Supplementary Table 5B.
Key to the roosts recorded as occupied by ≥100,000 *P. alecto* and *P. poliocephalus* during the Austral winter in each year from 1997 through 2020 and shown in Extended Data Figure 9 (Supplementary Table 5A, Dataset J[32]).

Key	Roost Name	State
A	Craignish Heights	QLD
B	Woocoo NP	QLD
C	Gympie	QLD
D	Kandanga	QLD
E	Indooroopilly	QLD
F	Woodend	QLD
G	Lismore Currie Park	NSW
H	Lismore Rotary Pk	NSW
I	Bingara	NSW
J	Tamworth	NSW
K	Stewarts Brook	NSW
L	Millfield	NSW
M	Morisset	NSW
N	Kioloa	NSW
O	Batemans Bay	NSW

was recorded. Mass flowering of that species at 3- to 5-year intervals supports flying fox roost populations that can exceed 200,000 for >3 consecutive months[55]. From these observations we hypothesized that highly productive pulses of winter flowering may moderate the risk of spillover and gathered historical data to assess this hypothesis.

There currently is no remote method available for monitoring temporal and spatial variations in eucalypt flowering at scale, although advances are being made[63]. We used the size of roosts occupied by BFF and GHFF as an index of nectar production and defined a highly productive pulse of flowering as a resource sufficient to support an estimated population of ≥100,000 flying foxes at a single roost. We set this threshold based on the unambiguous association between roosts this size and highly productive flowering pulses[14]; and the substantial influence roosts this size have on overall bat distribution and foraging patterns. When aggregations of ≥100,000 occurred in individual roosts, animals within these aggregations accounted for 34% to 63% of the total population, estimated within synchronous censuses. Only 0.09% of 1,563 winter roost counts from 1997-2019 were ≥100,000 whereas 92% of roost counts were <10,000. The results described here were not sensitive to a change in the threshold to 80,000. We then used winter roost census records from 1997 to 2020 acquired from various sources (see Data Index[7]) to identify flower pulses on the basis of roost populations ≥100,000 and estimate monthly start and duration (Dataset J[32]). This work enabled us to 1) explore overlap between the putative date of spillover (14 days prior to the date of death listed in Dataset A[12]) and flowering pulses (Fig. 2C), 2) document the location of winter aggregations (Supplementary Table 5A-B, Extended Data Fig. 9) and 3) identify candidate diet species either known to be in flower at the time large aggregations formed based on apiary data and records of field ecologists, or likely to have been in flower at the time based on their seasonal phenology and distribution[16].

We scaled our study to the mobility of nomadic flying foxes and monitored winter aggregations throughout southeast Australia (latitude -24.6 to -38.8). We were unable to source data on roost size in winter in six years, 1999, 2003, 2004, 2006, 2008 and 2014 (Supplementary Table 5), and identified five years in which either no roosts ≥100,000 were reported or aggregations of that size persisted for <2 weeks of the 3-month winter period, 2007, 2011, 2013, 2015 and 2017 (Fig. 2C). We identified seven species of winter-flowering diet plants that potentially contributed food resources in vegetation surrounding roosts at the time of large aggregations (in our study area: *E. robusta, E. siderophloia, E. tereticornis Melaleuca quinquenervia* and *Banksia integrifolia; E. albens* on the inland slopes of NSW; and *C. maculata* in the Hunter Valley of NSW and the NSW south coast; Extended Data Fig. 9). Over the 24-year study, large winter aggregations formed at 15 roosts (Dataset J[32], Extended Data Fig. 9, Supplementary Table 5). Eight of the roosts were located in our study area. The most distant was approximately 600 km south of the nearest study area boundary.

A subset of these data, restricted to roosts in the SEQ study area, was used to describe changes in the incidence of large winter aggregations in this region during the study period. These large aggregations were observed to be frequent between 1996-2002 but rare between 2007-2020 (Dataset C[27], Extended Data Fig. 5F).

12. Change in winter habitat in far southeast Queensland (SEQ) 1996–2018

To explore associations between environmental change and changes in bat behaviors we described loss of winter foraging habitat in the SEQ study area where long-term monthly roost censuses could be used to document behavioral change (Supplementary Information Section 5,

Extended Data Figure 4, Dataset C[27]). We identified five diet species that flower in this area during the winter bottleneck period: *Melaleuca quinquenervia*, *Eucalyptus robusta*, *E. siderophloia*, *E. tereticornis* and *Banksia integrifolia*. Using Queensland Regional Ecosystems (RE) vegetation classifications[72], modelled pre-clearing RE maps, Statewide Landcover and Trees Study (SLATS) land use data[73], and remotely sensed high-resolution (30m pixel) Global Forest Cover data[74] (Datasets K-M[7]), we mapped the distribution and area (ha) of this key winter habitat prior to European settlement and annually from 1996-2018. We generated maps of the distribution of habitat pre-clearing, at the start of the study period in 1996, and at the end of this study in 2018. Replacement land cover for each pixel of cleared land was assigned to one of seven broad categories in the SLATS data: crop, infrastructure, mine, natural disaster damage, pasture, settlement, thinning. These assignations were used to calculate the percentage of the total habitat cleared during the study that could be attributed to urban expansion or agricultural intensification.

All spatial data was projected to WGS84 Australian Albers Equal Area Conic projection for analyses and clipped to the study area boundary.

12.1 Identify key winter bottleneck habitat

We used the uniform vegetation classification for Queensland, Regional Ecosystems (RE), to describe the distribution of the five winter diet species[72], and identified key winter habitat as REs in which at least one of the five occurs as a dominant in the upper stratum.

12.2 Describe area and distribution of key winter habitat pre-clearing

To describe the distribution and area (ha) of key winter habitat prior to European settlement, we removed from the map of modelled pre-clearing RE data all vegetation that did not contain the winter diet plants as dominants. We then calculated the area of each remaining RE and generated a map of the distribution of winter habitat pre-clearing (Dataset K, see Data Index[7], Extended Data Fig. 5D).

12.3 Base map of winter habitat 1996

To examine annual patterns of loss in key winter habitat from 1996 to 2018, we first created a base map of the habitat extant in 1996. We used remotely sensed high-resolution (30m pixel) Global Forest Cover data to generate a map of forest cover in 2000, the only year in which full coverage of forest cover was available (Dataset L, see Data Index[7]). The data reports percentage canopy cover for vegetation of height >5m within each 30m pixel. We filtered the data for pixels of <15% canopy cover. This removed from the data small clusters of trees in urban settings which we considered unlikely to represent remnants of pre-clearing vegetation. Larger areas of non-native vegetation in horticultural plantings and plantation forests (primarily pine plantations) were identified from Queensland SLATS land use data (Dataset M, see Data Index[7])[73] and were also removed.

We acquired remotely sensed 30m pixel data of woody vegetation clearing across Queensland recorded in the SLATS land use data bi-annually 1995-1996 to 1998-1999, and then annually to 2018. We allocated total bi-annual clearing values in the 1995-1996 and 1998-1999 data evenly across each of the two years.

To define forest cover at the start of our study period in 1996, we merged clearing data from 1996 to 2000 and added those pixels to the 2000 forest cover data to create a raster of forest cover in 1996. The raster data was intersected with pre-clearing distribution of winter bottleneck REs to produce a base map of the distribution of key winter habitat in 1996.

12.4 Loss of key winter habitat 1996-2018

To describe annual loss of key winter habitat from 1996-2018 we intersected each set of annual clearing data with pre-clearing key winter habitat data; then progressively removed pixels cleared in each year to calculate annual area loss (ha). We calculated (1) percentage of pre-clearing habitat that remained each year from 1996 to 2018 (Extended Data Fig. 5E), (2) cumulative area cleared (Extended Data Fig. 5H), and (3) annual rate of loss as a percentage of the habitat that remained in the previous year (Extended Data Fig. 5G). We fit a smooth loess function to the latter, with a single outlier excluded (2003) to visualize trends in rates of annual clearing across years. Finally, we generated maps of the distribution of habitat at 3 time points: pre-clearing, at the start of the study period in 1996, and at the end of this study in 2018 (Extended Data Fig. 5D).

Replacement land cover for each pixel of cleared land was assigned in the SLATS data to one of seven broad categories: crop, infrastructure, mine, natural disaster damage, pasture, settlement, thinning. These assignations were used to calculate the percentage of the total habitat cleared during the study that could be attributed to urban expansion (combined settlement and infrastructure = 65%) or agricultural intensification (combined pasture, crop and thinning = 33%).

13. Multiscale Bayesian Network Model

Bayesian networks can learn and display conditional dependence between variables. Rather than pre-specifying a network structure, network models can identify and visualize relationships between variables. The network structure displays variables as nodes, and the edges, or lines as connecting nodes. The variables that are not connected are conditionally independent, whereas, variables with edges connecting them are related. Mathematically, a network model assesses the joint likelihood of the variables and determines whether the data supports a factorization that implies conditional independence. Practically, this factorization and the associated network are a visual representation of the mechanism of the scientific process.

Estimating a Bayesian network model consists of learning the graphical structure of the network and estimating the conditional probability distributions. Given that our focus is on prediction, we evaluate models with a Leave-One-Out (LOO) cross-validation approach[75]. In particular, the loo package[76] in R[77] is used to evaluate the Expected Log pointwise Predictive Density (ELPD) of candidate network models.

The multiscale and longitudinal structure of our collection of datasets violates independence assumptions and prohibits using existing software to learn the network structure or estimate conditional probabilities. There is a multiscale structure where some variables are recorded at the roost level, while others have a larger spatial range. Additionally, occupied roosts are included for multiple years over the course of the study. Hence, we developed a multiscale Bayesian network model to appropriately handle the structure of the data and evaluate network structures and learn conditional probability distributions of the variables[34].

13.1 Data Overview

We developed the hypothesis that the combination of climate-driven food shortages, land use, and winter flowering pulses are predictive for spillover, and derived six variables that represent these processes from key data: Oceanic Niño Index (ONI), food shortage, roost fissioning, land use within roost foraging area, a winter flowering pulse, and spillover at a roost (Supplementary Information

Fig. 1). We acknowledge that variables we are unable to measure also have influence, such as the susceptibility of recipient hosts, horses. However, we hypothesize that the variables we have identified are sufficient to identify and predict periods of high risk, which we define by clusters of 3 or more spillovers in a single winter, and periods of very low risk, which we define as winters with no or one spillover.

We hypothesize that the impact of climate on food shortages operates with a one-year lag and the impact of food shortages on spillover operates with a further one-year lag. Land use and spillover are local and the other variables are global.

Maximum Annual Oceanic Niño Index (ONI): ONI is a global measurement that we mapped into a single binary variable, for each year, to identify strong El Niño events $\geq$ 0.8 (Supplementary Information Section 6).

Food shortage: A binary variable for at least one month identified as a period of food shortage during a given year (Supplementary Information Section 7).

Roost fissioning: Roost fissioning is the number of new roosts established during a given year (Supplementary Information Section 4).

Land Use at Roost: Land use at the roost level is a composite variable derived from satellite imagery. To distinguish foraging areas that were less likely to contain horses (i.e. densely forested or densely urban) from those more likely to contain horses (i.e. more agricultural or a mosaic of land use types), we used thirty-meter resolution maps to quantify the portion of the roost's foraging area that can be classified as three categorical responses (Supplementary Information Section 9, Dataset I[34]). These three categories included: *forest* with over 75% of the foraging area as forested, *urban* with over 25% of the foraging area as built, and a final category that captures mosaic *agricultural* and lower-density, built environments (Fig. 3). The final results are not sensitive to small departures (~10%) from these threshold values between land use categorization.

Winter flowering pulse: A binary variable for a sustained pulse of native flowering during winter of a given year assessed by aggregations of ≥100,000 bats at a single roost (Supplementary Information Section 11).

Source roost of spillover: Locations of spillovers are established (Dataset A[12]) and allocated to the nearest roost (Supplementary Information Section 10). Data are counts of spillovers associated with each roost occupied by *P. alecto* in each winter. The final results are not sensitive to this allocation process.

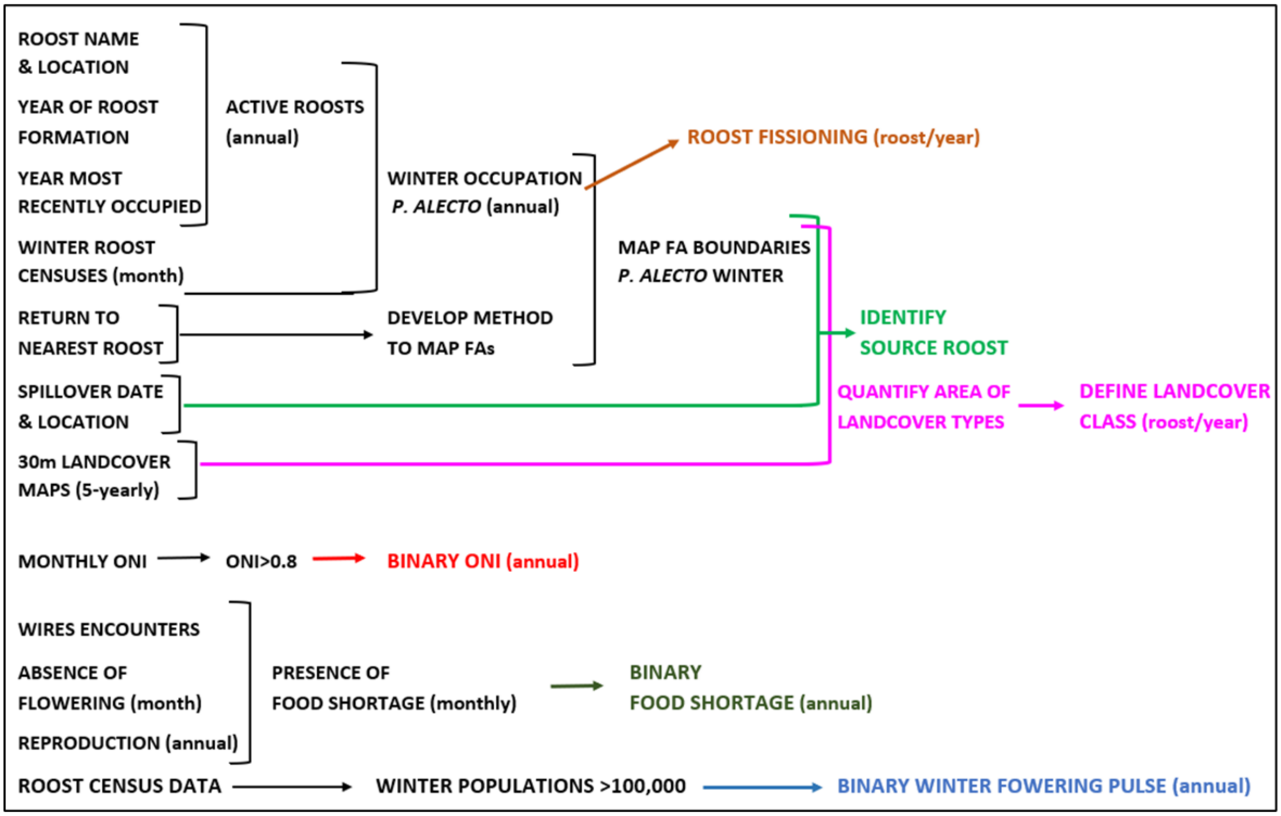

Supplementary Information Figure 1. Six variables (in colors) hypothesized to predict Hendra virus spillover were derived from 12 sets of data (left hand column) according to this flow diagram. Variables are described in Supplementary Information Sections 1-11.

13.2 Model Specification

Given the multiscale and longitudinal nature of the data, we describe a series of statistical probability distributions that appropriately handle this unique data structure and compose the joint probability distribution for the Bayesian networks. The responses for our set of variables are primarily binary or categorical, but roost counts also have discrete values. Binomial and multinomial probability distributions are used for binary and categorical data, while a negative binomial probability distribution is used for the discrete counts. Despite the moniker, Bayesian networks do not actually conduct fully Bayesian inference; to do so, we specify prior probability distributions on parameters of interest.

To evaluate a set of Bayesian network models requires computing probability distributions for each variable conditional on other parameters in the network. To evaluate a network, probability distributions are fit for each variable and ELPD is calculated for the full joint conditional distribution. Networks with the largest ELPD values are preferred.

Binomial Data: With a binary response and no longitudinal measurements across sampling units, a Bernoulli distribution can be used to model the probability of the binary outcome. This distribution is used for modeling food shortage and ONI, both of which are annual variables that apply globally to the entire study area.

In particular, let y_i be the binary outcome, θ is the probability of a success, and a and b are parameters in the prior distribution for θ.

$$y_i \sim \text{Bernoulli}(\theta) \quad (1)$$

$$\theta \sim \text{Beta}(a, b) \quad (2)$$

For these analyses both a and $b=1$.

Multinomial Data: Similar to the binary case, for a categorical response with no longitudinal measurements across sampling units, a multinomial distribution can be used. Given that winter flowering pulse contains positive values, negative values, and unknowns, there are three possible outcomes. Hence, a multinomial distribution allows us to simultaneously model the probability of all three categories.

Now let y_i be a vector that denotes whether the *ith* response was in category 1, 2, or 3 and θ is vector of probabilities that correspond to categories 1, 2, and 3.

$$y_i \sim \text{Multinomial}(\underline{\theta}) \quad (3)$$

$$\underline{\theta} \sim \text{Dirichlet}(\kappa \times \underline{\varphi}) \quad (4)$$

The prior distribution on $\underline{\theta}$ contains a concentration parameter κ and a mean parameter for the probabilities, $\underline{\varphi}$. For these analyses $\kappa = 1$ *and* $\underline{\varphi = (1/3, 1/3, 1/3)}$.

Hierarchical Binomial Data: When considering spillover at the roost level, the same roost will be included for multiple years. To account for the heterogeneity in spillover probability for a roost, we introduce a hierarchical binomial model that accounts for including the same roost multiple times. Specifically, each roost will have a roost-level spillover probability θ_i, which come from a hierarchical distribution that governs the spillover probability from all of the roosts.

Let y_{ij} be a binary variable to denote whether there is a spillover for the *jth* trial at the *ith* roost. As in[14], a hierarchical distribution for θ_i is set with a Beta distribution with mean ω and dispersion κ.

$$y_{ij} \sim \text{Bernoulli}(\theta_i) \quad (5)$$

$$\theta_i \sim \text{Beta}(\omega(\kappa - 2) + 1, (1 - \omega)(\kappa - 2) + 1) \quad (6)$$

$$\omega \sim \text{Beta}(a_\omega, b_\omega) \quad (7)$$

$$\kappa \sim \text{Gamma}(a_\kappa, b_\kappa) \quad (8)$$

Finally, prior distributions are specified for ω and κ, where $a_\omega = 0.1$, $b_\omega = 10$, $a_\kappa = 50$, *and* $b_\kappa = 0.5$.

Hierarchical Multinomial Data: The final scenario that we encounter in this analysis for categorical data is modeled with a hierarchical multinomial distribution. In this situation, land use has three possible outcomes, so a multinomial distribution is used, which is similar to the hierarchical binomial setting with longitudinal measurements for the sets of roosts.

Let y_{ij} be the vector of the response for the *jth* instance of the *ith* sampling unit. The vector $\underline{\theta}_i$ contains a vector of probabilities for each class for roost *i*.

$$y_{ij} \sim \text{Multinomial}(\underline{\theta}_i) \quad (9)$$

$$\underline{\theta}_i \sim \text{Dirichlet}(\kappa \times \underline{\varphi}) \quad (10)$$

$$\underline{\varphi} \sim \text{Dirichlet}(k_{\underline{\varphi}} \times \underline{p}) \quad (11)$$

$$\kappa \sim \text{Gamma}(a, b) \quad (12)$$

Using the same parameterization as the multinomial model, we encode the Dirichlet distribution with a mean vector κ and a concentration parameter $\underline{\varphi}$, both of which have a prior distribution. For these analyses $k_{\underline{\varphi}}=1$, $\underline{p} = (1/3, 1/3, 1/3)$, $a = 50$, and $b = 0.5$

Negative Binomial Data: When considering the number of new (fissioned) roosts in a year a negative binomial distribution is used. Let y_i be the number of new roosts in year *i*. We use a parameterization of the negative binomial that uses a mean and dispersion parameter

$$y_i \sim \text{Negbinomial}(\mu, \varphi) \quad (13)$$

$$\mu \sim \text{Gamma}(a, b) \quad (14)$$

$$\varphi \sim \text{Gamma}(a_\varphi, b_\varphi) \quad (15)$$

13.3 Fitting Multiscale Networks

Learning Graphical Structure: In exploring the relationships between the set of variables there are directional relationships that do not make sense with respect to time. For instance, a directional relationship from food shortage (previous year) to ONI values (2 years prior) does not make sense, but the opposite direction would be permitted. Supplementary Table 6 contains the edges that are restricted from the graphical structure.

A total of 64 plausible networks structures[34] were compared. The combination of LOO and ELPD compares the predictive ability of the model, by fitting the model on all the sampling units except the one left out for that cross-validation fold.

Supplementary Table 6: The edges of the directional relationships listed below are restricted in the graphical structure of the Bayesian Network Model as they are nonsensical.

FROM	TO
Winter Flowering Pulse	ONI (2 years prior)
Food Shortage (Previous Year)	ONI (2 years prior)
Winter Flowering Pulse	Food Shortage (Previous Year)
Food Shortage (Previous Year)	Winter Flowering Pulse

Estimating Conditional Probabilities: The network encodes the conditional probability structure between the variables, which maps to the mechanistic process to predict Hendra virus spillover. Based on this network, the probability of a spillover at a given roost in a given year would directly depend on whether there was a flowering pulse that year, a food shortage the previous year, and the land use type for the foraging area associated with the roost.

Simulation Test of Model Selection Procedure: We tested the reliability of our model selection procedure in a simulation study. Using our selected model (Model 36), we generated >1,000 data sets that were similar to the real data (same number of observations, etc.) but stochastic realizations of the model, then used our automated modelling procedure to fit Multiscale Bayesian Networks to these simulated data, tallied the number of times the true model is selected, investigated which other models may also be selected, and documented the distribution of Delta LOOIC. The simulation mimics the structure of the dataset with the same number of years and the same number of roosts in a given year. The estimated model parameters are used to simulate all other components of the dataset: land use type, elevated Oceanic Niño Index (ONI), presence of a food shortage, presence of a winter flowering pulse, number of new fissioned roosts, and spillover from a roost. The conditional dependence structure of the model identified using the methods above is used for the simulation.

Cross-validation Test of Predictive Capabilities: We tested the capabilities of our model to predict high and low risk conditions for spillover clusters using cross-validation. We randomly selected a portion of the dataset to remove from analysis and then used the rest of the dataset to make predictions for the held-out set. We used 5-fold cross validation to assess roost-year combinations in a specific fold. Predictions were made from a model fit on the roost-year combinations in the other four folds. The predictions of roost-year combinations were combined on an annual basis to calculate the predicted probability of a cluster of spillovers.

13.4 Model Results

We assessed a total of 64 different network models[34]. Leave one out cross validation results for the eight models most supported by the data are shown in Supplementary Table 7. The models were evaluated using Expected Log Pairwise Density (ELPD), where larger values indicate a better model. Note ELPD can be converted into an information criteria metric, Leave-One-Out Information Criteria (LOOIC), by multiplying by negative two.

LOOIC is on the same scale as other information criteria, such as AIC, BIC, DIC, or WAIC. Despite being on the same scale as AIC, using cross-validation, as with LOOIC can minimize overfitting relative to traditional IC methods as the predictive density is being evaluated on a data point not used to fit the model.

Supplementary Table 7 includes a sorted rank of the models with 10 LOOIC units from the best model. According to ELPD, and LOOIC, Model 36 is the best model, while the data also supports Model 40. The difference between Model 36 and Model 40 is the inclusion or exclusion of a connection between land use and spillover (Extended Data Fig. 6A). To a lesser extent, the data supports Model 33, which mimics Model 36 but has an additional connection between ONI and winter flowering. To make a determination of the best model, we interrogated the top two potential networks.

Supplementary Table 7: Leave one out cross validation results for top 8 models in the Bayesian Network analyses sorted by decreasing the Expected Log Pairwise Predictive Density (ELPD) / increasing Leave-One-Out information Criteria (LOOIC).

Model	ELPD	LOOIC
M36	-630.1	1260.2
M40	-631.1	1262.1
M33	-631.5	1263.0
M38	-632.2	1264.5
M34	-632.5	1264.9
M53	-633.4	1266.9
M42	-634.9	1269.7
M4	-635.0	1270.0

Estimation for network models is generally thought of as two distinct parts: fitting the network structure and estimating model parameters within a given network structure. In Model 36, the three-way interaction between land use, winter pulse, and food shortages resulted in a total of 18 parameters that corresponded to the probability of spillover as a combination of food shortage, winter flowering, and land use type. In Model 40, the two-way interaction between winter pulse and land use resulted in 6 parameters. Hence, by maintaining the same network structure, the complexity of these models was reduced substantially.

On the basis of our long-term data, we developed the parsimonious hypothesis that the combination of food shortages with the lack of winter flowering will result in the highest risk of spillover, especially in areas where land use supports agriculture. It was not as clear whether spillover risk would differ appreciably across other combinations of food shortage and winter flowering. Hence, we considered collapsing some of the levels of combinations across these three variables.

For Model 36 we considered more parsimonious models. The first contained two model parameters, a spillover probability for the food shortage, no winter spillover, and agricultural areas and a spillover probability for everything else. The second model contained four model parameters, a spillover probability for food shortage and winter spillover for each of the three land use types and a fourth spillover probability for everything else. With model 40 we evaluated a reduced model with no parameters that considers spillover probability for food shortage and no winter flowering and another spillover probability for everything else.

Supplementary Table 8 shows that all of the reduced models improved ELPD / LOOIC values relative to the values for the full models for the specified network structures. Both of the models with 2 parameters had similar LOOIC values. The reduced model for network structure 36 with 4 parameters had the best metric, with a value more than 2 units larger than competing models. While there was support for the 2-parameter models, the 4-parameter model for network structure 36 was a substantial improvement.

Supplementary Table 8: **Leave one out cross validation results for reduced models from the Bayesian Network analyses,** collapsing some of the interaction terms. Differences in leave-one-out information criteria (LOOIC) are shown for models with reduced parameter spaces as well as the other top 8 models.

**Model**	**Δ LOOIC**
M36: 4 parameters	0.00
M36: 2 parameters	1.98
M40: 2 parameters	5.85
M36	7.67
M40	9.57
M33	10.47
M38	11.97
M34	12.37
M53	14.37
M42	17.17
M4	17.47

Extended Data Figure 6B includes the distribution of LOOIC values (from M36) for each of the models from our simulation study. M36 is the favored model and at least the first quartile is positive for all other models. This model was selected as the best model using LOOIC 50 percent of the time (Supplementary Table 9). Model probabilities are shown for the models with the ten highest frequencies of having the lowest LOOIC values. The only other models with notable frequencies are M33, M40, and M60. The inclusion of M33 and M40 is unsurprising as these were the 2nd and 3rd strongest models according to LOOIC in our study (Extended Data Figure 6A). Each model differs from M36 by a single node. For M33 this is the addition of a connection from ONI to winter flowering pulse (a connection that could be investigated with further study) and for M40 this is the removal of the connection between land use and spillover. The difference in LOOIC between M36 and M40 is almost 6 and the difference between M36 and M33 is over 10, after collapsing unnecessary interactions in M36. M60, at least anecdotally, results from simulations where there is limited information available to estimate the joint impact of food shortages without winter flowering pulses (e.g. a single year out of 25 with winter flowering and food shortages, making it hard to estimate the relationship with little data).

Furthermore, we don't use LOOIC as a discriminating boundary when LOOIC between two models are relatively close as this would provide support for both models. In total, M36 is within a 2 LOOIC of the best model 70 percent of the time. In all 64 assessed Multiscale Bayesian Network models[34], we observe a difference in LOOIC of 5.85. From the simulations, M36 is within a 5.85 LOOIC of the best model 90 percent of the time.

Supplementary Table 9. Frequency of having the lowest leave-one-out information criteria (LOOIC) value across simulations. Top ten models are shown. M36 has substantially more support than any other model.

Frequency of Best LOOIC	Model
0.500	M36
0.132	M33
0.069	M40
0.061	M60
0.045	M53
0.044	M43
0.029	M56
0.026	M34
0.020	M49
0.015	M51

13.5 Sensitivity Analysis to Roost Assignment

Spillovers were assigned to the nearest roost; however, there was uncertainty in this process. To assess the impact of this we explored the implications of misspecification of the spillover-roost combination. With the modeling framework we are using, the total number of spillovers are aggregated by the combination of land use, winter flowering pulse, and food shortage. Given that food shortage and winter flowering pulse are global, and constant, across the study area for a specific year, we only needed to investigate the net impact on land use classification. For spillovers with a plausible second most likely roost, we explored the impact of shifting the attributions to the second roost. In total, this resulted in a net shift of one spillover in an urban area to an agricultural area. So even if all the roost assignments were mis-specified, the result would be minimal.

13.6 Stan Code for Model Fitting

The network model is fit using RStan[78,79]. Source stan code for each data type is provided below.

Binomial Data

```
data {
  int<lower=0> N;
  int<lower=0,upper=1> y[N];
  real<lower=0> a;
  real<lower=0> b;
}
parameters {
  real<lower=0,upper=1> theta;
}
model {
 theta ~ beta(a, b);
  y ~ bernoulli(theta);
}
```

```
generated quantities{
  vector[N] log_lik;
  for (n in 1:N) log_lik[n] = bernoulli_lpmf(y[n] | theta);
}
```

Multinomial Data

```
data {
  int<lower =1> M;
  int<lower=0> y[M];
  vector[M] phi;
  real<lower=0> kappa;
  int N;
  int<lower=0> y_mat[N, M];
}
parameters {
  simplex[M] theta;
}
model {
  theta ~ dirichlet(kappa * phi);
  // Count Data
  y ~ multinomial(theta);
}
generated quantities{
  vector[N] log_lik;
  for (n in 1:N) log_lik[n] = multinomial_lpmf(y_mat[n,] | theta);
}
```

Hierarchical Binomial Data

```
data {
  int<lower=0> M;
  int<lower=0> y[M];
  int<lower=0> N[M];
  real a;
  real b;
  real a2;
  real b2;
  int<lower=0> tot_obs;
  int group_index[tot_obs];
  int y_vec[tot_obs];
}
transformed data{
  real<lower=0> num_obs;
  num_obs = sum(N);
}
parameters {
  real<lower=0,upper=1> omega;
  real<lower=0> kappa;
  real<lower=0,upper=1> theta[M];
}
model {
  omega ~ beta(a, b);
  kappa ~ gamma(a2, b2);
  theta ~ beta(omega * (kappa -2) + 1, (1 - omega) * (kappa - 2) + 1);
  y ~ binomial(N, theta);
}
generated quantities{
   vector[tot_obs] log_lik;
   for (n in 1:tot_obs) log_lik[n] = bernoulli_lpmf(y_vec[n] |
theta[group_index[n]]);
}
```

Hierarchical Multinomial Data

```
data {
  int<lower=0> M;
  int<lower=1> K;
  int<lower=0> y[M, K];
  simplex[K] p;
  real k;
  real<lower=0> a;
  real<lower=0> b;
  int<lower=0> N;
  int<lower=0> y_mat[N,K];
  int<lower=0> group_index[N];
}
parameters {
  simplex[K] phi;
  simplex[K] theta[M];
  real<lower =0> kappa;
}
model {
 phi ~ dirichlet (k * p);
  kappa ~ gamma(a,b);
  for (i in 1:M) {
    theta[i,] ~ dirichlet(phi * kappa);
  }
  for (i in 1:M) {
    y[i,] ~ multinomial(theta[i,]);
  }
}
generated quantities{
  vector[N] log_lik;
  for (n in 1:N) log_lik[n] = multinomial_lpmf(y_mat[n,] |
theta[group_index[n]]);
}
```

Negative Binomial Data

```
data {
  int<lower=0> N;
  int<lower=0> y[N];
  real<lower=0> a;
  real<lower=0> b;
  real<lower=0> a_phi;
  real<lower=0> b_phi;
}
parameters {
  real<lower=0> mu;
  real<lower=0> phi;
}
model {
  mu ~ gamma(a, b);
  phi ~ gamma(a_phi, b_phi);
  y ~ neg_binomial_2(mu, phi);
}
generated quantities{
  vector[N] log_lik;
  for (n in 1:N) log_lik[n] = neg_binomial_2_lpmf(y[n] | mu, phi);
}
```

References

The following list contains references cited in Supplementary Information but not in the main text. Numbering continues from the main text reference list and references initially cited in the main text are indicated using the number assigned in that list.

48 Selvey, L. et al. Infection of humans and horses by a newly described morbillivirus. *Med J Aust* **162**, 642-645, doi.org:10.5694/j.1326-5377.1995.tb126050.x (1995).

49 Smith, C. et al. Twenty years of Hendra virus: laboratory submission trends and risk factors for infection in horses. *Epidemiol. Infect.* **144**, 3176-3183, doi.org:10.1017/S0950268816001400 (2016).

50 Middleton, D. et al. Hendra virus vaccine, a one health approach to protecting horse, human, and environmental health. *Emerg. Infect. Dis.* **20**, 372, doi.org:10.3201/eid2003.131159 (2014).

51 Manyweathers, J. et al. "Why won't they just vaccinate?" Horse owner risk perception and uptake of the Hendra virus vaccine. *BMC Vet. Res* **13**, 103, doi:10.1186/s12917-017-1006-7 (2017).

52 Annand, E.J. et al. Novel Hendra virus variant detected by sentinel surveillance of Australian horses. *Emerg. Infect. Dis.* **28**, 693–704, doi.org:10.3201/ eid2803.211245 (2022).

53 Wang, J. et al. A new Hendra virus genotype found in Australian flying foxes. *Virol J.* **18**, 197, doi:10.1186/s12985-021-01652-7 (2021).

54 Peel, A.J. et al. A novel variant of Hendra virus circulates in black flying-foxes (*Pteropus alecto*) and grey-headed flying-foxes (*Pteropus poliocephalus*), *Emerg. Infect. Dis.* **28**, 1043-1047, doi.org:10.3201/eid2805.212338 (2022).

55 Tidemann, C. Biology and management of the grey-headed flying-fox, *Pteropus poliocephalus. Acta Chiropt.* **1**, 151-164 (1999).

56 DAWE. National Recovery Plan for the Grey-headed Flying-fox '*Pteropus poliocephalus*', Department of Agriculture, Water and the Environment, Canberra, March. CC BY 4.0 (2021). https://www.environment.gov.au/biodiversity/threatened/recovery-plans.

57 Roberts, B. J. et al. Latitudinal range shifts in Australian flying-foxes: A re-evaluation. *Aust. Ecol.* **37**, 12-22, doi:10.1111/j.1442-9993.2011.02243 (2012).

58 Welbergen, J. et al. Extreme mobility of the world's largest flying mammals creates key challenges for management and conservation. *BMC Biology.* **18**, 101, doi.org/10.1186/s12915-020-00829-w (2021).

59 Fleming, P. S., Robinson, D. Flying-fox (Chiroptera Pteropodidae) on the north coast of New South Wales: Damage to stonefruit crops and control methods. *Aust. Mammal.* **10**, 143-145, doi.org:https://doi.org/10.1071/AM87032 (1987).

60 Clarke, M., Le Feuvre, D. Size and scope of the Australian honeybee and pollination industry. AgriFutures Australia. Publication No. 20-136. ISBN 978-1-76053-137-9 (2021).

61 Dowell, L. Observing the difference between flowering and non-flowering *Corymbia calophylla* (Marri) using satellite-derived vegetation indices. Business CRC (2018).

62 Somerville, D. Honey and Pollen Flora of South-eastern Australia. NSW Department of Primary Industries. ISBN 978-17605-83422 (2021).

63 Dixon, D.J. et al. Satellite prediction of forest flowering phenology. *Remote Sens. Environ.*, https://doi.org?10.1016/j.rse.2020.112197 (2021).

64 Somerville, D. Fat Bees Skinny Bees – honeybee nutrition for bee-keepers. Rural Industries Research and Development Corporation, Publication 05/054. ISBN: 1-74151-152-6 (2005).

65 Welbergen, J. A. et al. Climate change and the effects of temperature extremes on Australian flying-foxes. *Proc. Biol. Sci. B* **275**, 419-425, doi:10.1098/rspb.2007.1385 (2008).
66 Therneau, T. et al. Rpart: Recursive Partitioning. R Package Version 4.1-3. http://CRAN.R-project.org/package=rpart (2013).
67 Wakefield, E. D. et al. Space partitioning without territoriality in gannets. *Science* **341**, 68–70, doi: 10.1126/science.1236077 (2013).
68 Lourie, E. et al. Memory and conformity, but not competition, explain spatial partitioning between two neighboring fruit bat colonies. *Front. Ecol. Evol.* **9**, 732514, doi: 10.3389/fevo.2021.732514 (2021).
69 Baddeley, A, Turner, R. spatstat: An R Package for Analyzing Spatial Point Patterns. *J. Stat. Softw,* **12**, 1–42, doi: 10.18637/jss.v012.i06 (2005).
70 Wood, S. Generalized Additive Models: An Introduction with R, 2nd edition. Chapman and Hall/CRC (2017).
71 Calderón-Loor, M., Hadjikakou, M. & Bryan, B. A. High-resolution wall-to-wall land-cover mapping and land change assessment for Australia from 1985 to 2015. *Remote Sens. Environ.* **252**, 112148 (2021).
72 Queensland Herbarium. Regional Ecosystem Descriptive Database v11. https://web.archive.org/web/20190422210918/https://www.qld.gov.au/environment/plants-animals/plants/ecosystems/descriptions/download (2019).
73 Queensland Department of Environment and Science. Statewide landcover and trees study (SLATS): Overview of Methods (2018).
74 Hansen, M. C. et al. High-resolution global maps of 21st-century forest cover change. *Science* **342**, 850-853, doi:10.1126/science.1244693 (2013).
75 Vehtari, A., Gelman, A. & Gabry, J. Practical Bayesian model evaluation using leave-one-out cross-validation and WAIC. *Stat. Comput.* **27**, 1413-1432 (2017).
76 Vehtari, A. et al. Loo: Efficient leave-one-out cross-validation and WAIC for Bayesian models. R package version 2.3.1 (2020).
77 R Core Team. R: A language and environment for statistical computing. R Foundation for Statistical Computing, Vienna, Austria: Available at: https://www.R-project.org. (2020).
78 Kruschke, J. Doing Bayesian data analysis: A tutorial with R, JAGS, and Stan. (2014).
79 Stan Developent Team. RStan: the R interface to Stan R package version 2.21.2. http://mc-stan.org/ (2020).
