# Supplementary material for: Pathogen spillover driven by rapid changes in bat ecology
Source: Nature. 2022 Nov 16;613(7943):340–4. doi: 10.1038/s41586-022-05506-2 (PMC9768785; doi:10.1038/s41586-022-05506-2)
Supplement: Supplementary file 2 — Reporting Summary [file 41586_2022_5506_MOESM2_ESM.pdf]

nature portfolio

Corresponding author(s): Raina Plowright

Last updated by author(s): October 24th 2022

Reporting Summary

Nature Portfolio wishes to improve the reproducibility of the work that we publish. This form provides structure for consistency and transparency in reporting. For further information on Nature Portfolio policies, see our Editorial Policies and the Editorial Policy Checklist.

Statistics

For all statistical analyses, confirm that the following items are present in the figure legend, table legend, main text, or Methods section.

n/a	Confirmed	
☐	☒	The exact sample size (*n*) for each experimental group/condition, given as a discrete number and unit of measurement
☐	☒	A statement on whether measurements were taken from distinct samples or whether the same sample was measured repeatedly
☒	☐	The statistical test(s) used AND whether they are one- or two-sided<br>*Only common tests should be described solely by name; describe more complex techniques in the Methods section.*
☐	☒	A description of all covariates tested
☒	☐	A description of any assumptions or corrections, such as tests of normality and adjustment for multiple comparisons
☐	☒	A full description of the statistical parameters including central tendency (e.g. means) or other basic estimates (e.g. regression coefficient) AND variation (e.g. standard deviation) or associated estimates of uncertainty (e.g. confidence intervals)
☒	☐	For null hypothesis testing, the test statistic (e.g. *F*, *t*, *r*) with confidence intervals, effect sizes, degrees of freedom and *P* value noted<br>*Give P values as exact values whenever suitable.*
☐	☒	For Bayesian analysis, information on the choice of priors and Markov chain Monte Carlo settings
☐	☒	For hierarchical and complex designs, identification of the appropriate level for tests and full reporting of outcomes
☒	☐	Estimates of effect sizes (e.g. Cohen's *d*, Pearson's *r*), indicating how they were calculated

Our web collection on statistics for biologists contains articles on many of the points above.

Software and code

Policy information about availability of computer code

Data collection	no software was used
Data analysis	Data are analyzed with R (version 4.2.1), R packages: tidyverse, rpart, rattle, rstan, HDInterval, loo, bnlearn, ggridges, ggtern. Data also analyzed with Stan version 2.26.1. Code provided in Supplementary Information and in eCommons repository. Details are provided in Eby, Peggy, Alison Peel, Andrew Hoegh, Wyatt Madden, John Giles, Peter Hudson, and Raina Plowright (2022) Data and scripts from Pathogen spillover driven by rapid changes in bat ecology. Food shortage regression tree model [Dataset]. Cornell University eCommons Digital Repository. https://doi.org/10.7298/rdbe-cy49 and Eby, Peggy, Alison Peel, Andrew Hoegh, Wyatt Madden, John Giles, Peter Hudson, and Raina Plowright (2022) Data and figure from Pathogen spillover driven by rapid changes in bat ecology. Bayesian network model [Dataset]. Cornell University eCommons Digital Repository. https://doi.org/10.7298/y0nr-e545

For manuscripts utilizing custom algorithms or software that are central to the research but not yet described in published literature, software must be made available to editors and reviewers. We strongly encourage code deposition in a community repository (e.g. GitHub). See the Nature Portfolio guidelines for submitting code & software for further information.

Data

Policy information about availability of data

All manuscripts must include a data availability statement. This statement should provide the following information, where applicable:

- Accession codes, unique identifiers, or web links for publicly available datasets
- A description of any restrictions on data availability
- For clinical datasets or third party data, please ensure that the statement adheres to our policy

Data Availability

The datasets generated and analyzed during the current study are available in the Cornell University eCommons Digital Repository or they are available as open access files. The URLs are provided in the Data Index https://doi.org/10.7298/pjjb-3360, with the exception of records from commercial apiarists (Supplementary Information Section 7) that are constrained by commercial in-confidence considerations. Dataset URLs: Dataset A: https://doi.org/10.7298/3dbp-t721; Dataset B: https://doi.org/10.7298/kdht-sp38; Dataset C: https://doi.org/10.7298/ajmw-mp18; Dataset E: https://doi.org/10.7298/tb5p-dr98; Dataset F: https://doi.org/10.7298/j3q2-gw32; Dataset G: https://doi.org/10.7298/3vha-5m37; Dataset I: https://doi.org/10.7298/x71e-c660; Dataset J: https://doi.org/10.7298/rmhz-dc23.

Field-specific reporting

Please select the one below that is the best fit for your research. If you are not sure, read the appropriate sections before making your selection.

☐ Life sciences ☐ Behavioural & social sciences ☒ Ecological, evolutionary & environmental sciences

For a reference copy of the document with all sections, see nature.com/documents/nr-reporting-summary-flat.pdf

Ecological, evolutionary & environmental sciences study design

All studies must disclose on these points even when the disclosure is negative.

Study description	This work brings together multiple datasets that were collected over 25 years. The Data Index supplies a summary of datasets and how to access them: Eby, P. et al. Data from Pathogen spillover driven by rapid changes in bat ecology. Data Index. Cornell University eCommons Digital Repository. doi:https://doi.org/10.7298/pjjb-3360 (2022).
Research sample	Extensive data collection on spillover, flying fox population dynamics, flying fox movement, Oceanic Nino Index, apiary records, animal intakes into rehabilitation organisations, reproductive output, foraging data, foraging area characteristics (land cover and land use), winter flowering pulses (bat aggregations), habitat clearing. See Data Index (above)
Sampling strategy	n/a
Data collection	See Data Index for the sources of data (doi:https://doi.org/10.7298/pjjb-3360). The field data was collected by P. Eby.
Timing and spatial scale	See Data Index for the temporal and spatial scale associated with each dataset (doi:https://doi.org/10.7298/pjjb-3360).
Data exclusions	The extent of the study area was defined after the 2011 Hendra virus outbreaks in the subtropics. The study area encompassed all detected Hendra virus spillovers in the subtropics at that time, including the feeding areas of the associated flying fox roosts. This is described in the supplementary information and supplementary methods. Ongoing data collection was focused within this study area. See Supplementary Information.
Reproducibility	No experiments were performed.
Randomization	n/a
Blinding	n/a

Did the study involve field work? ☒ Yes ☐ No

Field work, collection and transport

Field conditions	Observations of reproductive output were performed during the same week each year; see supplementary methods and information. Census and population counts of flying fox populations were performed.
Location	Subtropical Australia, see Supplementary Table 2.
Access & import/export	n/a
Disturbance	n/a

Reporting for specific materials, systems and methods

We require information from authors about some types of materials, experimental systems and methods used in many studies. Here, indicate whether each material, system or method listed is relevant to your study. If you are not sure if a list item applies to your research, read the appropriate section before selecting a response.

Materials & experimental systems

n/a	Involved in the study
☒	☐ Antibodies
☒	☐ Eukaryotic cell lines
☒	☐ Palaeontology and archaeology
☒	☐ Animals and other organisms
☐	☒ Human research participants
☒	☐ Clinical data
☒	☐ Dual use research of concern

Methods

n/a	Involved in the study
☒	☐ ChIP-seq
☒	☐ Flow cytometry
☒	☐ MRI-based neuroimaging

Human research participants

Policy information about studies involving human research participants

Population characteristics	Participants in the nectar monitoring research met at least one of the following criteria:<br>1) apiarists that manage substantial enterprises (hundreds of hives); 2) active members of industry support groups (federal, state and local industry associations, government advisory personnel) and 3) honey packers to whom apiarists sell their products. No other characteristics were considered
Recruitment	Recruitment occurred in the following steps. Potential participants were identified via the recommendations of: office holders of federal, state and local industry support organizations, government advisory personnel, and existing participants. Researchers contacted potential participants directly to confirm they met criteria for participation (above) and agreed to provide information on nectar production as set out in the methods of the study. Finally, participants were required to provide informed consent under the Griffith University Human Research Ethics Committee. approval (below).
Ethics oversight	The Griffith University Human Research Ethics Committee (GUHREC) approved the study protocol (GU Ref No: 2022/765)

Note that full information on the approval of the study protocol must also be provided in the manuscript.
